# Multi-omics Analysis of Experimentally Evolved *Candida auris* Isolates Reveals Modulation of Sterols, Sphingolipids, and Oxidative Stress in Acquired Amphotericin B Resistance

**DOI:** 10.1111/mmi.15379

**Published:** 2025-06-04

**Authors:** Anshu Chauhan, Hans Carolus, Dimitrios Sofras, Mohit Kumar, Praveen Kumar, Remya Nair, Aswathy Narayanan, Kusum Yadav, Basharat Ali, Vladislav Biriukov, Amandeep Saini, Ian Leaves, Rudy Vergauwen, Celia Lobo Romero, Dhara Malavia-Jones, Ashutosh Singh, Atanu Banerjee, Shivaprakash M. Rudramurthy, Arunaloke Chakrabarti, Alok K. Mondal, Naseem A. Gaur, Kaustuv Sanyal, Jeffrey M. Rybak, Toni Gabaldón, Patrick Van Dijck, Neil A. R. Gow, Rajendra Prasad

**Affiliations:** 1 Amity Institute of Biotechnology, Amity University Gurugram, Haryana, India; 2 Laboratory of Molecular Cell Biology, Department of Biology, KU Leuven, Leuven, Belgium; 3 Leuven One-health Institute, Leuven, Belgium; 4 Winship Cancer Institute of Emory University, Decatur, Georgia, USA; 5 Jawaharlal Nehru Centre for Advanced Scientific Research, Bengaluru; 6 School of Life Sciences, Jawaharlal Nehru University, Delhi; 7 Institute for Research in Biomedicine (IRB), Barcelona Institute of Science and Technology, Spain; 8 Barcelona Supercomputing Centre (BSC-CNS), Spain; 9 Catalan Institution for Research and Advanced Studies (ICREA), Spain; 10 CIBER de Enfermedades Infecciosas, Instituto de Salud Carlos III, Spain; 11 Medical Research Council Centre for Medical Mycology at the University of Exeter, Geoffrey Pope Building, Stocker Road, Exeter, EX4 4QD, UK; 12 Department of Biochemistry, University of Lucknow, Lucknow; 13 Post-Graduate Institute of Medical Education & Research, Chandigarh; 14 Yeast Biofuel Group, International Centre for Genetic Engineering and Biotechnology, New Delhi; 15 Department of Pharmacy and Pharmaceutical Sciences, St. Jude Children’s Research Center, USA

**Keywords:** fAMR, amphotericin B, oxidative stress, sphingolipids, sterols

## Abstract

Clinical isolates of *Candida auris* show a high prevalence of resistance to Amphotericin B (AmB) - an uncommon trait in most *Candida* species. Alterations in ergosterol biosynthesis can contribute to acquired AmB resistance in *C. auris* laboratory strains but are rarely seen in clinical isolates. In this study, we experimentally evolved two drug-susceptible Clade II isolates of *C. auris* to develop AmB resistance. The evolved strains displayed a 4 to 8-fold increase in MIC_50_ compared to the parental cells. We analyzed changes in their karyotype, genome, lipidome, and transcriptome associated with this acquired resistance. In one lineage, *AOX2* was upregulated, and its deletion reversed the AmB resistance phenotype. The *aox2Δ* mutant also failed to evolve AmB resistance under experimental conditions. In the same lineage, restoring the *UPC2*^*S332R*^ *and RTG3*^*S101T*^ mutations to the wild-type allele restored AmB susceptibility. In another lineage, the ergosterol and sphingolipid pathways were observed to play a critical role, and upregulation of the ERG genes elevated the total sterol content, while significant downregulation of *HSX11* (glucosylceramide synthase) resulted in lower levels of glucosylceramides. To our knowledge, this study is the first to show that AmB resistance in *C. auris* can be acquired through mechanisms both dependent or independent of sterol content modulation, highlighting Aox2 and Upc2 as Key regulators of amphotericin resistance.

## Graphical abstract

**Graphical abstract:**
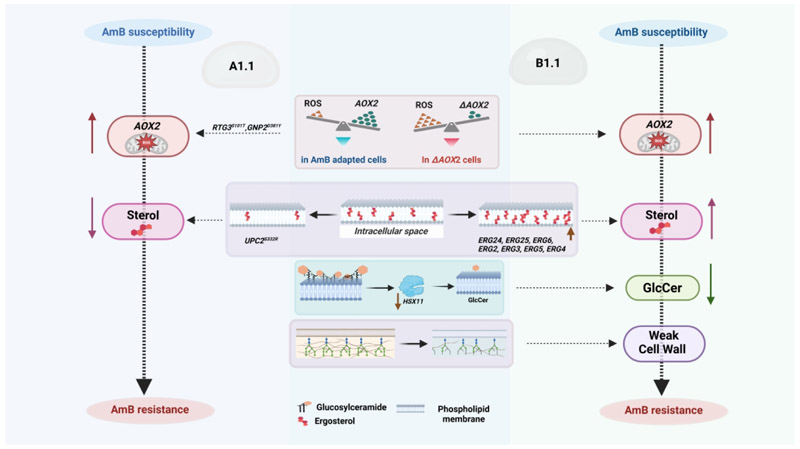
An overview of resistance mechanisms in two East Asian *C. auris* isolates evolved in vitro. The A1.1 strain acquired resistance by enhancing oxidative stress responses through the *AOX2, RTG3*, and *GNP2* genes, along with sterol-related modifications driven by *UPC2*. In contrast, the B1.1 strain developed resistance through changes in glucosylceramide synthesis, cell wall integrity, and the ergosterol biosynthesis pathway. Together, highlighting both sterol-dependent and sterol-independent mechanisms contributes to AmB resistance.

## Introduction

*Candida auris* (recently reassigned to the genus *Candidozyma* and renamed as *Candidozyma auris* (Liu *et al*., 2024) was first identified as a human pathogen when it was isolated from the ear canal of a female patient in Japan in 2009 (Satoh *et al*., 2009). Following this discovery, phylogenetically distinct strains of *C. auris* were subsequently isolated from patients in several other countries, often in patients with bloodstream infections (Calvo *et al*., 2016; Lockhart *et al*., 2017; Ben-Ami *et al*., 2017; Borman *et al*., 2017; Schwartz and Hammond, 2017; Abastabar *et al*., 2019; Iguchi *et al*., 2019; Chow *et al*., 2020; CDC, 2024). Retrospective analyses of samples revealed that this species may have been present in clinical settings earlier but were misidentified due to their similarity to other species of *Candida* (Kathuria *et al*., 2015). The near-simultaneous emergence of multidrug-resistant *C. auris* across multiple continents has become a global concern. *C. auris* poses unique challenges compared to other pathogenic *Candida* species, particularly due to its resistance to multiple classes of antifungal agents (Sharma and Chowdhary, 2017; Sharma and Chakrabarti, 2020) and its elevated stress resistance, making it difficult to decontaminate hospital surfaces. Currently, four major classes of antifungal drugs are used to treat invasive fungal infections: azoles, polyenes, echinocandins, and nucleic acid analogs (Rogers and Barker, 2003; Cowen *et al*., 2015). Almost all *C. auris* isolates are resistant to fluconazole, approximately 50% are resistant to voriconazole, 30% to amphotericin B (AmB), and 10% to echinocandins (Sekyere, 2017; Rybak *et al*., 2022b).

Polyenes such as AmB exert their antifungal effects by extracting ergosterol from the fungal cell membrane (Lewandowska *et al*., 2021). The AmB forms sponges in the cell wall that remove ergosterol from the cell membrane, leading to its destabilization. (Anderson *et al*., 2014). Additionally, increased expression of *ERG1, ERG2*, and *ERG6* has been linked to AmB resistance, suggesting a compensatory response to counteract the disruption caused by AmB's interaction with ergosterol (Young *et al*., 2003; Vincent *et al*., 2013; Muñoz *et al*., 2018a; Ahmad *et al*., 2019; Silva *et al*., 2020).

Several mechanisms of acquired AmB resistance have been described in the literature. Clinically acquired AmB resistance was initially caused by a frameshift mutation in the *ERG6* gene, leading to premature truncation of Erg6 in patients, causing high resistance of *C. auris* to AmB (Rybak *et al*., 2022a). Additionally, Kordalewska *et al*., 2021 reported that a truncated 164 amino acid Erg6 protein conferred AmB resistance. Mutations in, *NCP1, ERG11, ERG3, HMG1, ERG10*, and *ERG12*, have been linked to AmB resistance in *C. auris* (Carolus *et al*., 2024), and the loss-of-function of *ERG6*, resulting in the production of cholesta-5,7,24-trienol instead of ergosterol, is the most commonly identified mechanism of AmB resistance in experimentally evolved *C. auris* strains (Carolus *et al*., 2024). In other *Candida* species, mutations in *ERG1, ERG2*, and *ERG6* genes have also been linked to AmB resistance (Ahmad *et al*., 2019; Carolus *et al*., 2020). Beyond sterol biosynthesis, DNA damage checkpoint proteins, such as Mec3, and the transcription factor Flo8, which positively regulates *ERG11* expression, have also been suggested to play a role in AmB resistance in *C. auris* (Escandón *et al*., 2019; Carolus *et al*., 2021b).

To investigate the molecular basis of AmB resistance further, we experimentally evolved two susceptible East Asian isolates of *C. auris* from Clade II at sub-lethal concentrations of AmB over 100 generations. The AmB resistant cells maintained their higher minimum inhibitory concentrations (MICs) after serial passage in drug-free media, suggesting that resistance was due to stable inheritable mutations. Subsequent functional analysis of these mutants using genomic, transcriptomic, and lipidomic analyses highlighted the contributions of *AOX2* and *UPC2* and modulations of the sterol and sphingolipid pathways in the acquired AmB resistance phenotype.

## Results

### Clade identification of the selected isolates

Strain P2428 (NCCPF 470296) was isolated from a diabetic patient at PGIMER, Chandigarh, India. PCR products that were produced using Clade-specific primers (Figures S1a and S1b) (Narayanan *et al*., 2022b) and the raw WGS reads (explained in the Materials and methods section) were subjected to sequencing analysis for clade identification as depicted in the phylogenomic analysis (Figure S1c), which showed that it was a Clade-II isolate. Strain B11220 (CBS10913^T^) is the type strain of Clade II (Satoh *et al*., 2009; Muñoz *et al*., 2018b) and was re-confirmed as a Clade II isolate in our analysis (Figure S1a). This analysis suggests that *C. auris* clades other than Clade I are circulating in the Indian subcontinent. The two parental strains, B11220 and P2428, were designated as wt_A and wt_B, respectively.

### Experimental evolution of *C. auris* susceptible isolates

**Figure 1:**
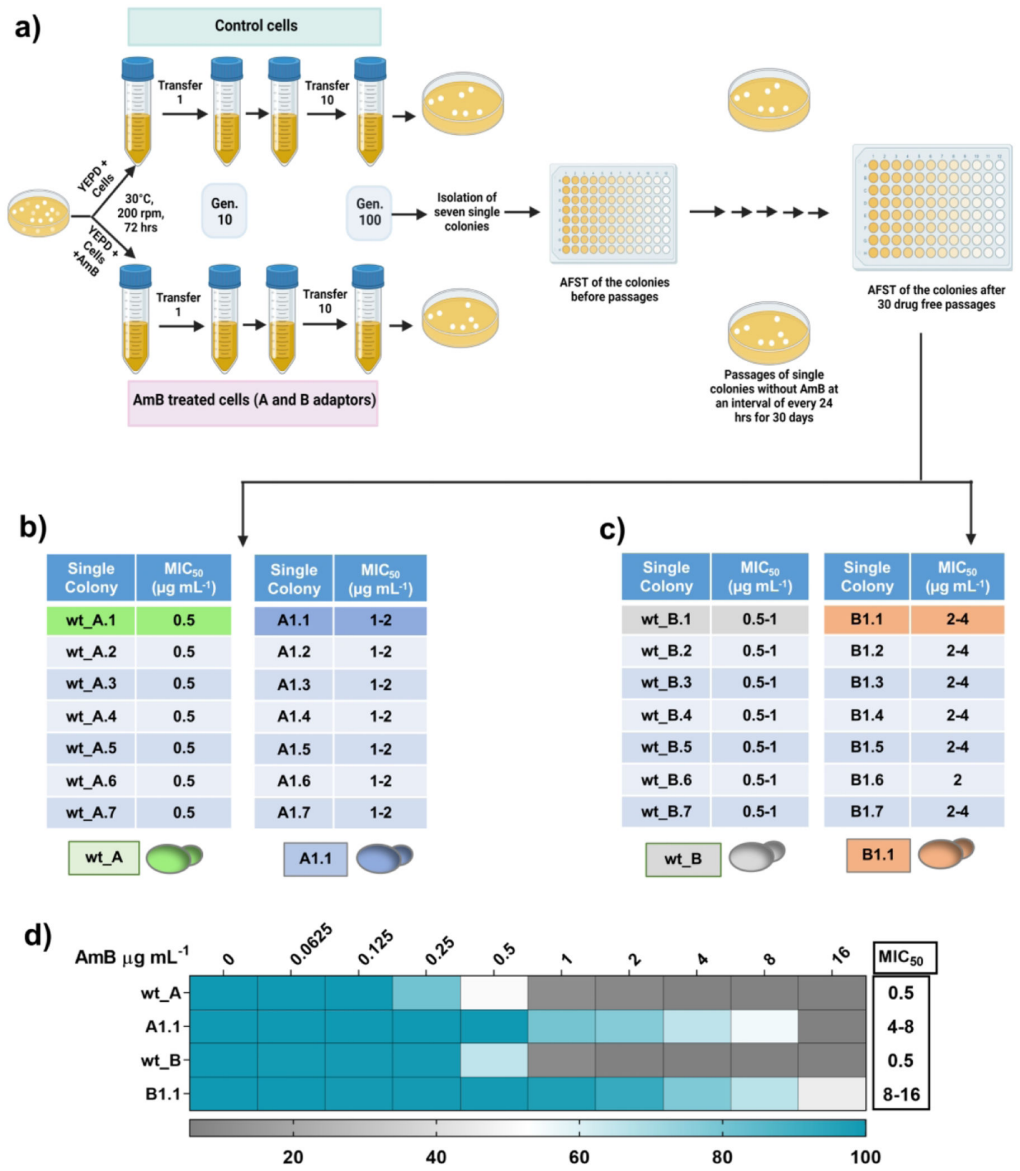
Experimental evolution and evolvability of a *C. auris* strain toward AmB resistance. **a)** Schematic depiction of the *in vitro* microevolution regime followed in this study. **b) and c)** Selection of single colonies from the adapted replicates of parental strain wt_A and wt_B, respectively, displaying high resistance to AmB retained after the drug-free passages. **d)** MIC_50_ values of the final colonies termed as A1.1 and B1.1 were determined by broth dilution assay. The MIC_50_ values in RPMI medium were observed to be lower (for A1.1, 2 µg mL^-1^, and for B1.1, 2-4 µg mL^-1^) but beyond the breakpoint for AmB, implying the cells to be resistant towards AmB.

Single colonies from strain wt_A (MIC_50_ value of 0.5 µg mL^-1^ for AmB) and strain wt_B (MIC_50_ value 0.5–1 µg mL^-1^ for AmB) were subjected to experimental evolution by exposing each of them to AmB concentration equivalent to their MIC_50_ values for 100 generations (Figure 1a) (for details, see Material and Methods). After 100 generations, evolved strains were collected. A single colony from each evolved strain of wt_A and wt_B was selected for further analysis, and they were referred to as A1 and B1, respectively. Strains A1 and B1 were subjected to AmB susceptibility testing using microdilution assays. Both adapted strains exhibited 4-8-fold higher MIC_50_ values compared to the parent strains.

We assessed whether these AmB-resistant adapted strains, A1 and B1, retained the acquired resistance under non-selective conditions. This was tested by passaging single colonies from each adapted replicate for 30 days on drug-free agar medium. After 30 days of daily passage, more than 25 single colonies isolated from adapted strains A1 and B1 were tested for AmB resistance, and one single colony from each A1 and B1, designated A1.1 and B1.1 adapted strains, was used for further analysis (Figures 1b and 1c). The adapted strains A1.1 and B1.1 exhibited MIC_50_ values of 2 µg mL^-1^ and 4 µg mL^-1^, respectively, for AmB (Figure 1d). The A1.1 and B1.1 adapted strains, when tested for acquired resistance to other antifungals, were found to exhibit cross-resistance to other polyenes, nystatin, and natamycin. However, no cross-resistance to azoles was observed (Figures S2, and S3). There were varying levels of cross-resistance to other polyenes in both adapted strains. While A1.1 exhibited resistance to nystatin and natamycin similar to that of AmB, B1.1 showed slightly higher resistance to nystatin than to natamycin (Figure S2). Both strains displayed increased MIC_50_ values, yet remained susceptible to azoles. A1.1 stayed within the susceptibility range but showed a 2-fold increase in MIC_50_ for fluconazole, voriconazole, and clotrimazole compared to wt_A. B1.1 showed minimal changes for most azoles, except for a comparable 2-fold MIC_50_ increase for fluconazole and ketoconazole (Figure S3). These results indicate that enhanced polyene resistance due to adaptation did not compromise azole susceptibility.

### Whole genome sequencing (WGS) analysis revealed novel SNPs

WGS of A1.1 and B1.1 adapted strains revealed six SNPs, including missense and nonsense mutations in the A1.1-adapted strain and three missense mutations in the B1.1-adapted strain (Table 1).

**Figure 2:**
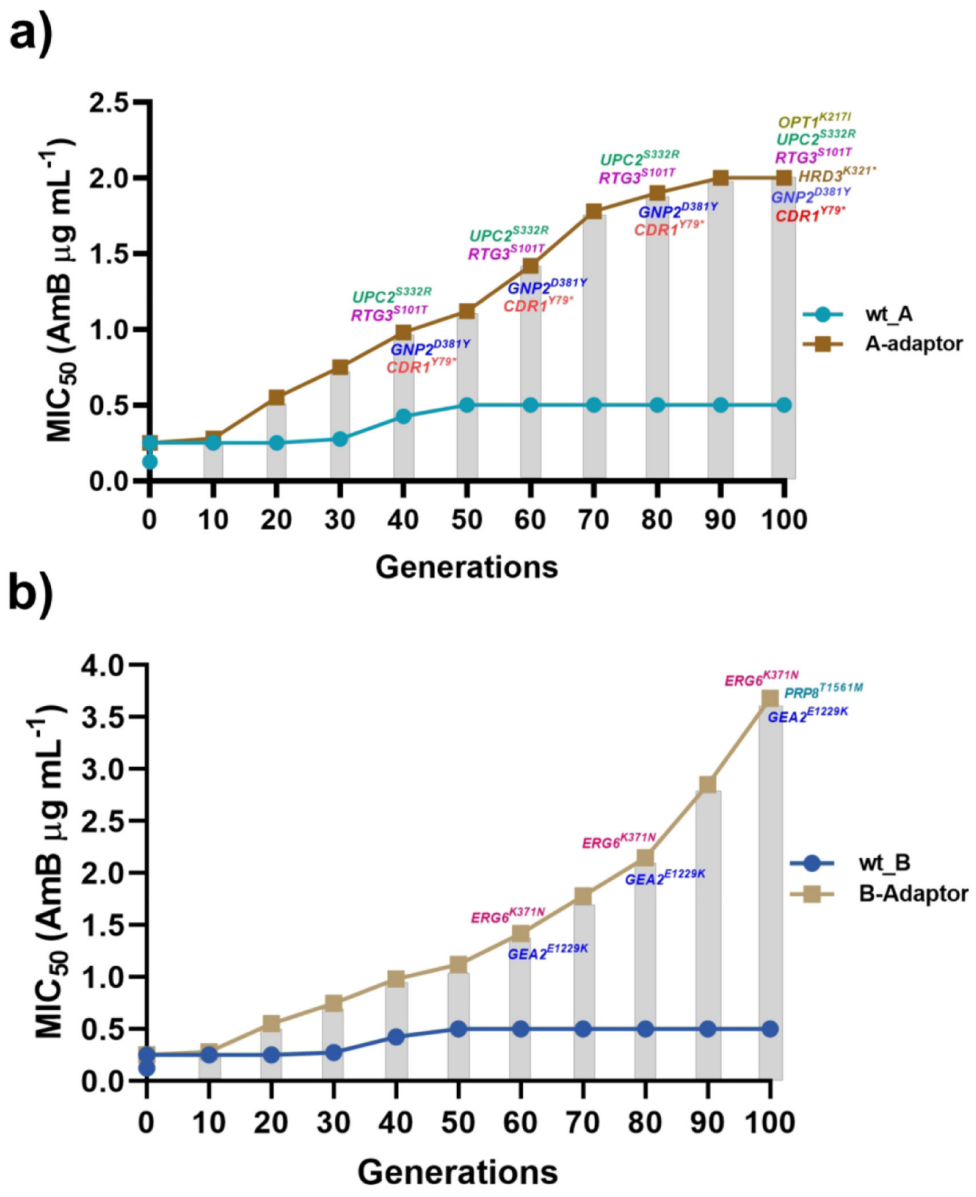
The appearance of the SNPs in the course of AmB resistance evolution. **a)** the presence of SNPs in the adapted strain A1.1, and **b)** depicts the appearance of SNPs in the adapted strain B1.1 during the course of adaptation towards AmB. The grey bars represent a progressive increase in MICs.

The emergence of these SNPs largely coincided with the observed increase in MICs toward AmB. For instance, the targeted sequencing of adapted isolates from intermediate generations revealed that in the A1.1 adapted strain, where increased MIC_50_ value towards AmB was recorded from generation 50 onwards, out of six SNPs, *UPC2*^*S332R*^, *RTG3*^*S101T*^, *GNP2*^*D381Y*^, and *CDR1*^Y79*^ appeared from generation 40 onwards. The only exceptions were those of *OPT1*^*K217I*^ and *HRD3*^K321*,^ which emerged only after generation 80 (Figure 2a). In the B1.1 adapted strain, two of the three SNPs (*ERG6*^*K371N*^ and *GEA2*^*E1229K*^) emerged by generation 60 onwards, while *PRP8*^*T1561M*^ was detected after generation 80 (Figure 2b). Collectively, this mid-point analysis of the SNPs in the adaptors during the evolution experiments highlights a strong correlation between the appearance of specific SNPs and the increased AmB MICs.

Changes in ploidy associated with AmB resistance in micro-evolved *C. auris* strains have been reported (Carolus *et al*., 2024). However, no significant changes were identified in karyotype and genome coverage analyses of the A1.1 and B1.1 adapted strains in the present study (Figure S4).

### The significance of the mutations in *UPC2, RTG3, GNP2 and ERG6* in the adapted strains

**Figure 3:**
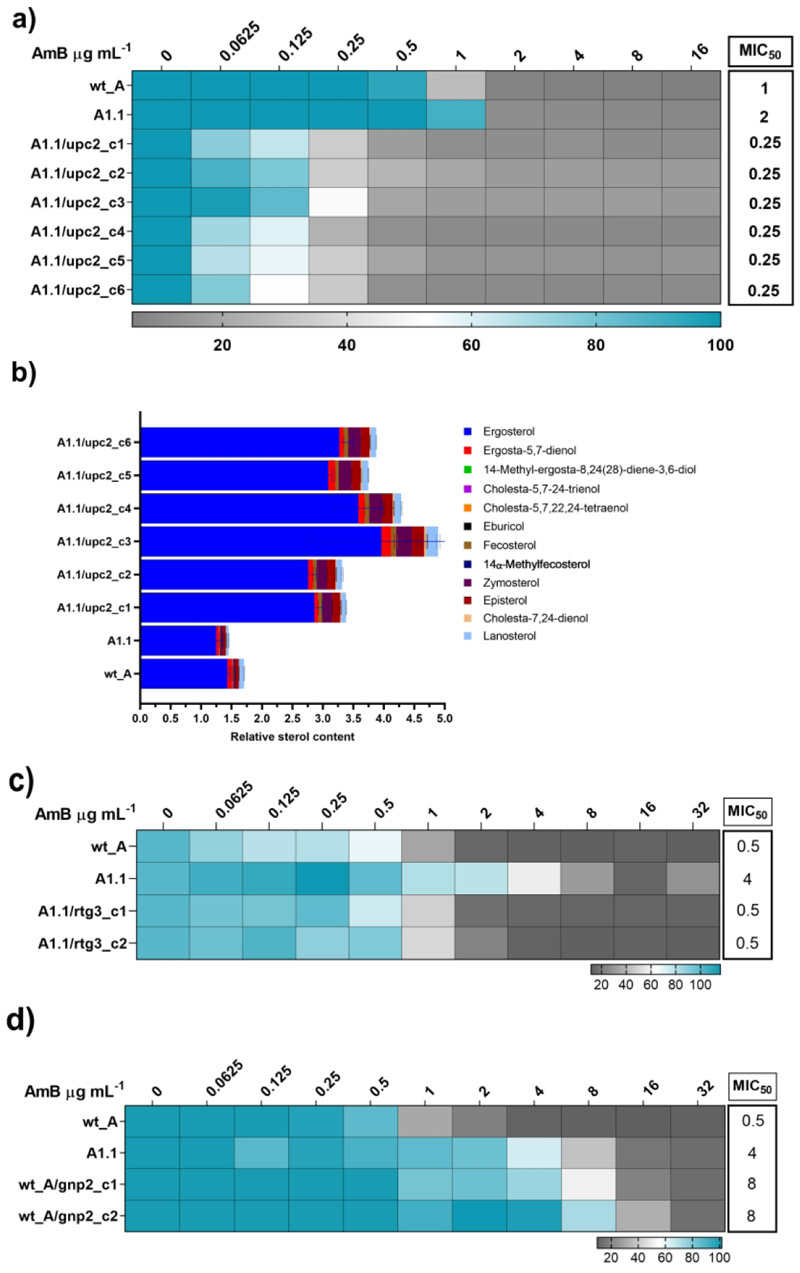
Impact of *UPC2*^*S332R*^ on AmB susceptibility and sterol levels. **a)** Effect of the point mutation *UPC2*^*S332R*^ restoration to the wild type sequence resulting in increased susceptibility of the strains to AmB. **b)** Total sterol content in the *UPC2* SNP sequence-restored colonies in comparison to the wt_A and the mutant adapted strain A1.1. The sterol content represented in the figure is relative to the internal standard. **c)** *RTG3*^*S101T*^ mutation reversal increased susceptibility to wt_A level. **d)** introduction of the *GNP2*^*D381Y*^ SNP in the wt_A strain resulted in increased resistance to AmB, like in the adapted strain.

Among the SNPs observed in the A1.1 adapted strain, the one in the *UPC2* gene, which encodes a transcription factor, looked interesting as this mutation, *UPC2*^*S332R*^, is upstream of the hotspot of gain-of-function mutations (Flowers *et al*., 2012) and has not been reported previously. To investigate the contribution of this mutation to AmB resistance, we restored the mutation harbored by the A1.1 adapted strain by employing a *C. auris* optimized Episomal Plasmid Induced Cas9 (EPIC)-mediated gene editing system (Sofras *et al*., 2025; Doorley *et al*., 2025). The transformation by EPIC plasmid yielded six colonies (designated as A1.1/upc2_c1-c6), which upon subsequent validation by PCR and gene sequencing, confirmed that all the six positive transformants had a restored mutation to the native allele sequence (the guide RNA sequences utilized in this study are provided in Table S3). All these colonies showed decreased resistance to AmB implying that the restoration of the *UPC2*^*S332R*^ mutation to the native allele also reversed AmB resistance (Figure 3a). Since *UPC2* regulates sterol metabolism in yeast, we performed sterol profiling of the transformed colonies. All the colonies with the restored native *UPC2* sequence (A1.1/upc2_c1-c6) also showed a significant increase in ergosterol levels (Figure 3b). Reverting the *UPC2*^*S332R*^ mutation to the wild-type allele in the A1.1 background resulted in approximately a 10-fold increase in susceptibility to AmB, accompanied by elevated ergosterol levels. While the observation is intriguing, this could plausibly be a result of the underlying genetic heterogeneity between the SNP-reverted adapted strains (A1.1/upc2_c1–c6) and the original wild-type strain (wt_A). The A1.1 strain carries multiple SNPs (Table 1) that are absent in wt_A and could contribute to the observed phenotype. Although preliminary, these findings provide the first direct genetic evidence linking the *UPC2*^*S332R*^ mutation to altered AmB susceptibility.

The other identified SNPs included mutations in the transcription factor encoding gene, *RTG3*, and the high-specificity proline permease encoding gene, *GNP2*. To assess their impact on AmB susceptibility, we reverted the *RTG3*^*S101T*^ mutation to the wild-type allele and introduced *GNP2*^*D381Y*^ into the wild-type strain. Reversion of *RTG3*^*S101T*^ restored AmB susceptibility in the A1.1 adapted strain (Figure 3c) (the guide RNA sequences utilized in this study are provided in Table S3). Interestingly, the wild-type strain harboring the *GNP2*^*D381Y*^ mutation (*wt_A/GNP2*^*D381Y*^) displayed greater resistance to AmB than the adapted A1.1 strain. This is likely attributable to differences in genetic background: A1.1 carries multiple additional mutations, whereas *wt_A/GNP2*^*D381Y*^ contains only the single introduced mutation. Despite the variation in MIC_50_ between the two strains, these findings underscore the significant role of the *GNP2*^*D381Y*^ mutation alone in conferring AmB resistance in *C. auris*.”

The *ERG6*^*K371N*^ mutation was one of three SNPs identified in the B1.1 adapted strain (Table 1). This specific mutation in *ERG6* had not been previously linked to AmB resistance in *C. auris*. In this study, the sterol profile of the B1.1 strain carrying the *ERG6*^*K371N*^ mutation revealed an accumulation of cholesta-5,7,24-trienol along with the substantial level of ergosterol (Figure 8b). These findings suggest that *ERG6*^*K371N*^ may represent a partial loss-of-function mutation, leading to the buildup of this intermediate sterol. Therefore, *ERG6*^*K371N*^ could contribute, at least in part, to the development of AmB resistance, as has also been observed earlier (Carolus *et al*., 2024).

### Global transcriptomic analysis of AmB-resistant adaptors reveals a variable number of differentially expressed genes (DEGs)

**Figure 4:**
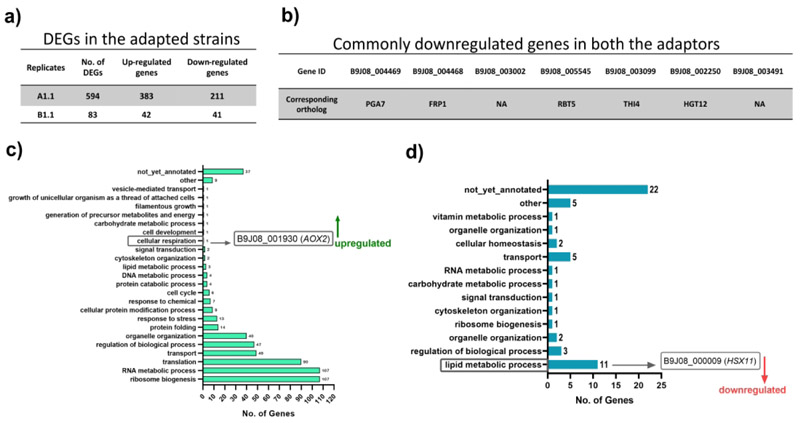
Transcriptomic profile of the adapted strains A1.1 and B1.1. **a)** The total number of differentially expressed genes (DEGs), the upregulated and downregulated genes in A1.1 and B1.1 strains. **b)** the commonly downregulated genes in the adapted strains, A1.1 and B1.1. **c)** and **d)** the Gene Ontology enrichment analysis depicting the various cellular processes associated with the DEGs. The up-regulation of *AOX2* and down-regulation of *HSX11* are highlighted.

To understand the molecular basis of the increased resistance to AmB in the evolved strains, we performed temporal global transcriptomic profiling of adapted lines. We used a 1.5-log_2_ fold change in the expression as the threshold for the analysis of DEGs with an associated *p-value* of ≤0.05 as significant for our analysis. The global transcriptome of A1.1 and B1.1 adapted strains revealed a variable number of DEGs among the replicates (Supplementary sheets S1 and S2). The total number of DEGs in A1.1 and B1.1 were 594 and 83, respectively. Among these, the upregulated genes were 383 and 42 in A1.1 and B1.1, and 211 and 41 were downregulated in A1.1 and B1.1, respectively (Figure 4a). Notably, there were no common upregulated genes found in both the adapted strains, but there were seven common downregulated genes in these strains (Figure 4b). Gene Ontology enrichment analysis revealed that among the DEGs linked to various cellular processes, an elevated level of an uncharacterized gene, *B9J08_001930*, which is an orthologue of the *AOX2* gene in *C. albicans* and encodes an alternative oxidase *AOX2*, and down-regulation of *HSX11* were most noteworthy (discussed below).

### An alternative oxidase, Aox2 contributes to AmB resistance

**Figure 5:**
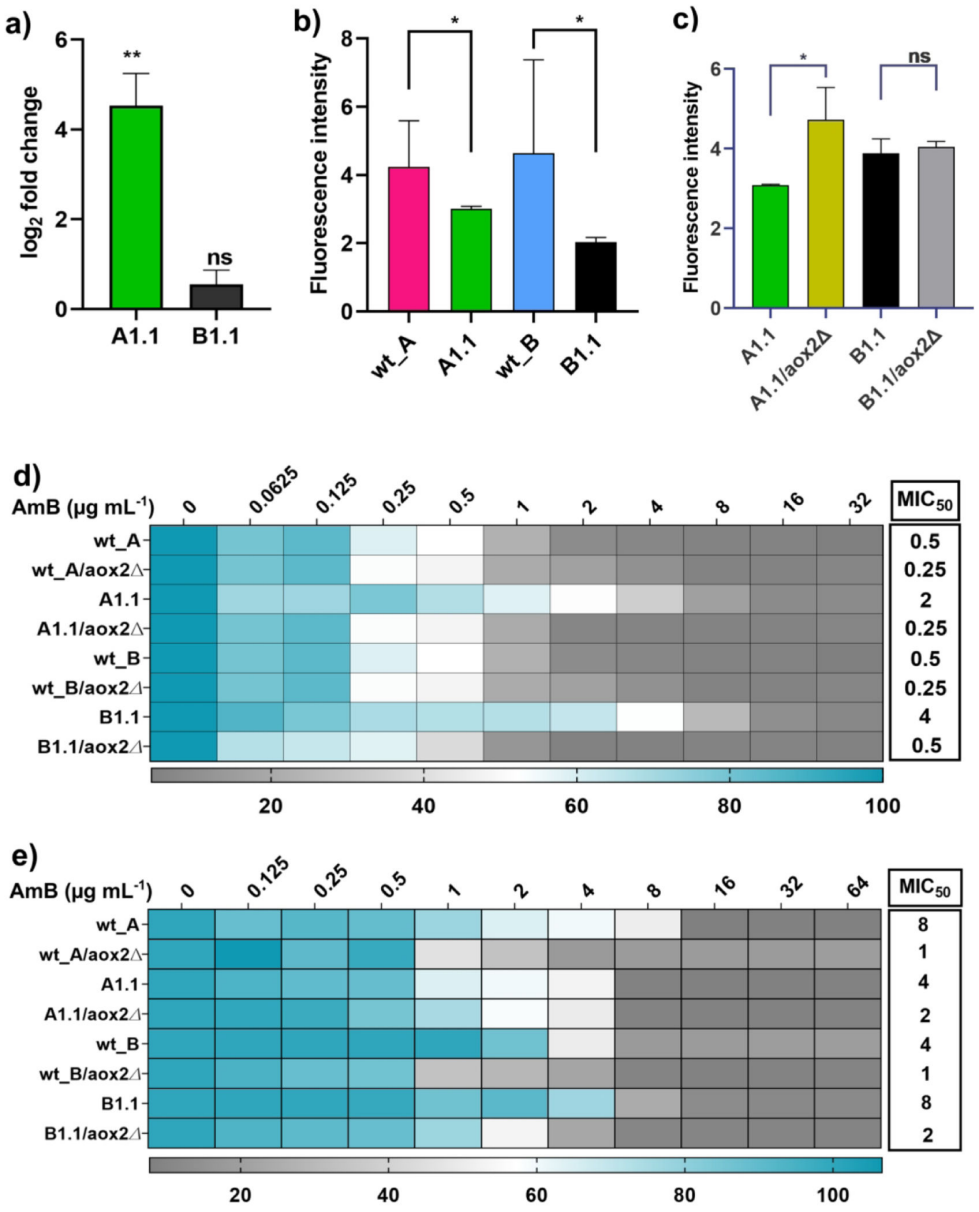
*AOX2* impacts AmB resistance. **a)** Real-time quantitative PCR showing differences in expression levels of the *AOX2* gene in the adapted strains, A1.1 and B1.1. The expression levels were normalized against the *C. auris* housekeeping gene *ACT1*. **b)** and **c)** The levels of ROS in the adapted strains and respective *AOX2* knockouts. The ROS levels were estimated by the fluorescent dye DCFH-DA (2'-7'-Dichlorodihydrofluorescein diacetate). All the experiments were performed in biological triplicates and technical duplicates. Bar graphs represent Mean ± SD (n=3) fluorescence intensity. **d)** Heatmap depicting the change in the MIC_50_ values of the adapted strains and their respective deletion strains. **e)** Heatmap depicting that *AOX2* mutants exhibit impaired adaptability to AmB resistance after completion of approximately 100 generations of microevolutionary experiment.

As described earlier, the transcriptome analysis revealed higher upregulation of *AOX2* in the A1.1-adapted strain compared to the B1.1-adapted strain. The log_2_-fold change value in the A1.1 adaptor was 8.8 log_2_-fold, and B1.1 showed a more modest 1.3 log_2_-fold increase compared to their respective wild-type progenitors. This was further confirmed by qRT-PCR, where the upregulation of *AOX2* in A1.1 adapted strain was a 5-6 log_2_-fold increase, and in B1.1 adapted strain, it was a 0.8-0.9 log_2_-fold increase (Figure 5a). Both A1.1 and B1.1 strains exhibited significantly reduced reactive oxygen species (ROS) levels, potentially due to the elevated *AOX2* expression (Figure 5b). Deletion of *AOX2* in both adapted strains (A1.1/*aox2Δ*, and B1.1/*aox2Δ*), as well as in their parental controls (wt_A, and wt_B), demonstrated *AOX2*’s role in AmB resistance. Specifically, the *AOX2* deletion mutants of the adapted strains displayed markedly increased susceptibility to AmB (Figure 5d). Consistent with this, ROS levels were elevated in A1.1/*aox2Δ* cells, though no comparable increase was observed in B1.1/*aox2Δ* mutants (Figure 5c). Interestingly, *AOX2* deletion in the wild-type progenitors (wt_A and wt_B) resulted in a marginal increase in susceptibility towards AmB (Figure 5d).

To further assess whether *AOX2* directly contributes to the acquisition of AmB resistance, we subjected *AOX2* knockout strains to experimental evolution under sublethal AmB pressure. Both wild-type strains (wt_A, and wt_B) and their respective *AOX2* deletion mutants (wt_A/*aox2Δ* and wt_B/*aox2Δ*) were evolved for 100 generations in the presence of AmB. Unlike the wild-type strains, which successfully evolved AmB resistance, the *AOX2* knockout strains failed to develop resistance, indicating a direct role for *AOX2* in facilitating adaptation to AmB (Figure 5e). Furthermore, when *AOX2* was deleted in the already adapted strains (A1.1 and B1.1), the resulting mutants (A1.1/*aox2Δ*, and B1.1/*aox2Δ*) retained only a limited capacity to adapt to AmB, showing reduced resistance compared to their parental adapted strains.

### *AOX2* influences AmB susceptibility in a clinical isolate of *C. auris* from clade I

**Figure 6:**
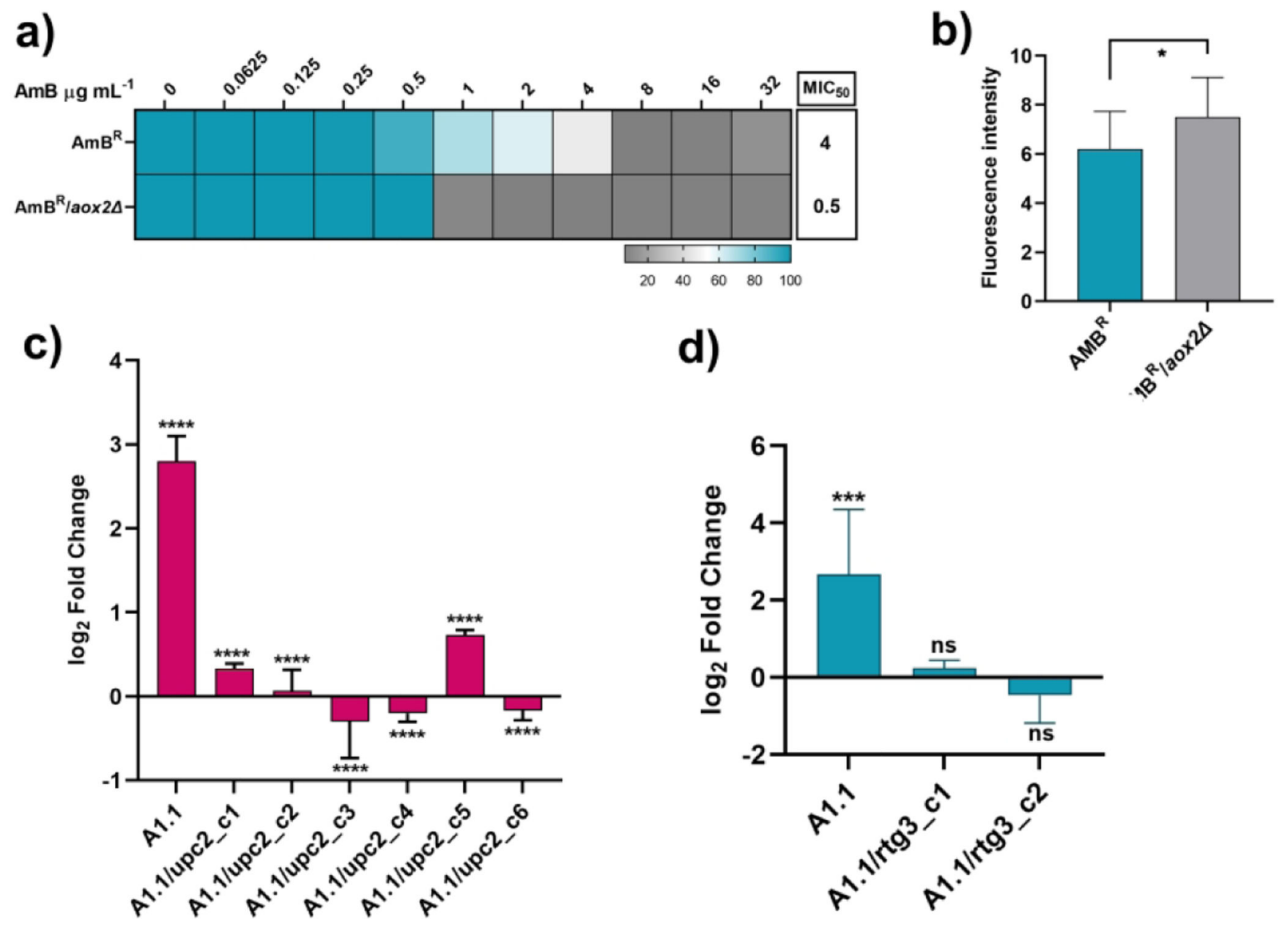
Role of *AOX2* in the AmB resistant clinical isolate of Clade I and effect of SNPs impact on expression levels of *UPC2*, and *RTG3* on *AOX2*. **a)** AmB susceptibility increases upon deletion of *AOX2* in the AmB^R^ clinical isolate. **b)** ROS levels were significantly increased in the *AOX2* knockout strain, AmB^R^/aox2Δ. **c)** The reversal of *UPC2*^*S332R*^ also reverted *AOX2* expression to the basal level, and **d)** the reversal of *RTG3*^*S101T*^ to wild type resulted in basal expression levels of *AOX2* as compared to the adapted strain A1.1.

Since our study is focused on clade II, which is not typically associated with human-invasive infections or outbreaks and shows lower pathogenicity in infection models, we also explored the relevance of *AOX2* in clinical AmB resistance. We deleted *AOX2* in an AmB-resistant (AmB^R^) clinical isolate of clade I (denoted as AmB^R^/*aox2Δ*), which resulted in an >8-fold decrease in AmB MICs (Figure 6a). As expected, AmB^R^/*aox2Δ* cells also exhibited increased ROS accumulation (Figure 6b). These findings underscore the general significance of *AOX2* in AmB resistance of clinical isolates. Collectively, our data suggest that *AOX2* plays a crucial role in modulating AmB susceptibility, not only in AmB-adapted strains but also in AmB-resistant clinical isolates.

### Aox2 expression is regulated by *UPC2* and *RTG3*

In the A1.1 adapted strain, SNPs were identified in two key transcription factors, Upc2 and Rtg3. Reverting these mutations to their wild-type alleles restored AmB susceptibility to levels similar to the progenitor strain. This prompted us to investigate whether these transcription factors also influence *AOX2* expression. Analysis of *AOX2* expression in the *UPC2* and *RTG3* SNP-reverted strains revealed that, unlike the A1.1 adapted strain where *AOX2* is highly upregulated, the reverted strains exhibited basal *AOX2* expression levels (Figure 6c, 6d). These results suggest that Upc2 and Rtg3 play a regulatory role in controlling *AOX2* expression. Notably, Rtg3-mediated regulation of *AOX2* appears to be conserved, consistent with a previous study by Liu et al., 2023 demonstrating that Rtg3 regulates *AOX2* promoter activity, impacting *C. albicans* virulence in a mouse model.

### Sphingolipids and ergosterol modulate AmB resistance

**Figure 7:**
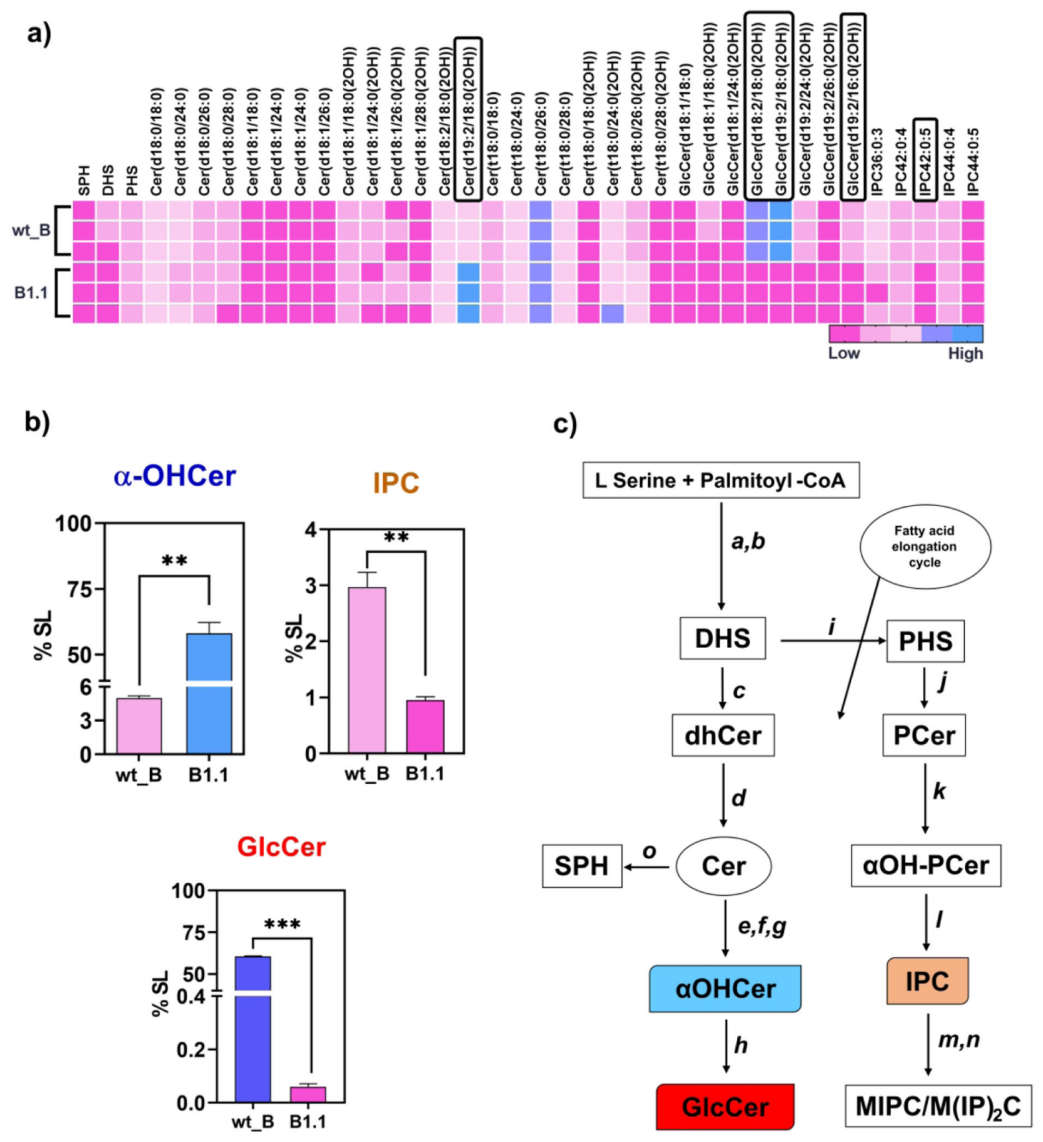
Sphingolipid-focused lipidomics analysis of B1.1 adapted strain. **a)** Heatmap representing the relative abundance of SL molecular species in the wild type (wt_B) and the adapted strain B1.1. The molecular species exhibiting highly significant differences are highlighted in boxes. **b)** Quantitative comparison of three SL classes that showed significant differences between wt_B and B1.1. Bar graphs represent %SL (Mean ± SEM of three biological replicates). Reduced levels of GlcCer were observed in B1.1 adapted strain, along with the accumulation of its precursor, α-OHCer. Additionally, IPC levels were also diminished. Significance was calculated using Student’s *t*-test. *** denotes p ≤ 0.0001, and ** represents p ≤ 0.001. **c)** Schematic representation of part of the SL biosynthesis pathway. The enzymes catalyzing various steps of the pathway, are a) serine palmitoyl transferase, b) 3-keto dihydro sphingosine reductase, c) ceramide synthases, d) Δ4-desaturase, e) α-hydroxylase, f) Δ8-desaturase, g) methyl transferase, h) GlcCer synthase (*HSX11*), i) C4-hydroxylase (LCB hydroxylase), j) ceramide synthase, k) α-hydroxylase, l) IPC synthase, m) MIPC synthase, n), M(IP)_2_C synthase, and o), ceramidase.

The DEG analysis showed that the *B9J08_000009* gene, which is an ortholog of *HSX11* in *C. albicans* encoding glucosylceramide (GlcCer) synthase, was among the highly downregulated genes (-4.33-log_2_ fold change) in the B1.1 adapted strain. Hsx11 catalyzes the transfer of UDP-linked glucose to the sphingoid backbone of ceramide precursors (Ali *et al*., 2024). We performed sphingolipid (SL)-focused lipidomic analysis, and our results identified a drastic reduction of GlcCer levels in the B1.1-adapted strain (<1%) compared to the wt_B cells (~60%). There was a concomitant accumulation of α-hydroxyceramides (α-OHCer), which are precursors of GlcCer. The levels of other SL biosynthetic intermediates did not significantly differ between B1.1 and wt_B cells (Figure 7a). The significant reduction in GlcCer was also accompanied by changes in several molecular species with different sphingoid backbones, such as d18:1, d18:2, and d19:2, and different fatty acid chains (Figure 7a). The major GlcCer contributing species was GlcCer (d19:2/18:0(2OH)), and its precursor Cer (d19:2/18:0(2(OH)) which accumulated in the B1.1-adapted strain (Figure 7a and supplementary file S3). Based on our earlier analysis, glucosyl derivatives are the main complex SLs found in *C. auris*, indicating that the GlcCer branch of the SL pathway is active in this species (Kumar *et al*., 2021). However, the specific role of GlcCer levels in drug resistance is not fully understood. Inositol phosphoryl ceramides (IPCs) are a class of complex anionic SLs found in fungi, plants, and some protozoans but are absent in mammals. They are characterized by the presence of an inositol group linked to the ceramide backbone at the C-1 position. In fungi, these are known to interact with sterols in the form of lipid rafts and are known to mediate multiple roles, including virulence (Shahi *et al*., 2020). Our lipid analysis of the B1.1 adapted strain could only quantify Inositol phosphoryl ceramide (IPCs) and observed their reduction in the adapted strain (Figures 7b and 7c). We also found that genes related to predicted oxidoreductase activity (*B9J08_000008*), carnitine acetyltransferase (*B9J08_000010*), and two uncharacterized genes (B9J08_000006 and *B9J08_000007*) were downregulated in the same scaffold PEKT02000001 where *HSX11* is present.

### *HSX11* levels do not directly impact AmB susceptibility

To further investigate the role of *HSX11*, we generated a knockout strain in the wt_B background, designated wt_B/*hsx11Δ*. Given that *HSX11* was strongly downregulated in the B1.1 adapted strain, we hypothesized that deleting *HSX11* in wt_B might mimic this condition. However, the wt_B/*hsx11Δ* strain showed no significant change in AmB susceptibility compared to the parental wt_B strain (Figure. S5). This unexpected outcome suggests that *HSX11* is not the primary or sole contributor to AmB resistance in the B1.1 adapted strain. It is also possible that the distinct genetic background of wt_B did not fully replicate the context of B1.1, potentially masking the impact of *HSX11* deletion on AmB susceptibility. For instance, in the B1.1 adapted strain, there is upregulation of ergosterol biosynthesis pathway genes, increased sensitivity to cell wall-perturbing agents, and altered expression of cell wall-related genes (discussed later). Moreover, the presence of an *ERG6* SNP leads to a partial loss-of-function mutation, as confirmed by the altered sterol profile of B1.1. The role of *ERG6* in mediating AmB resistance has been well documented in previous studies (Rybak *et al*., 2022a; Carolus *et al*., 2024). Taken together, these findings suggest that AmB resistance in the B1.1 strain results from a combination of factors, including enhanced sterol biosynthesis, compromised cell wall integrity, and reduced GlcCer levels due to *HSX11* downregulation.

**Figure 8:**
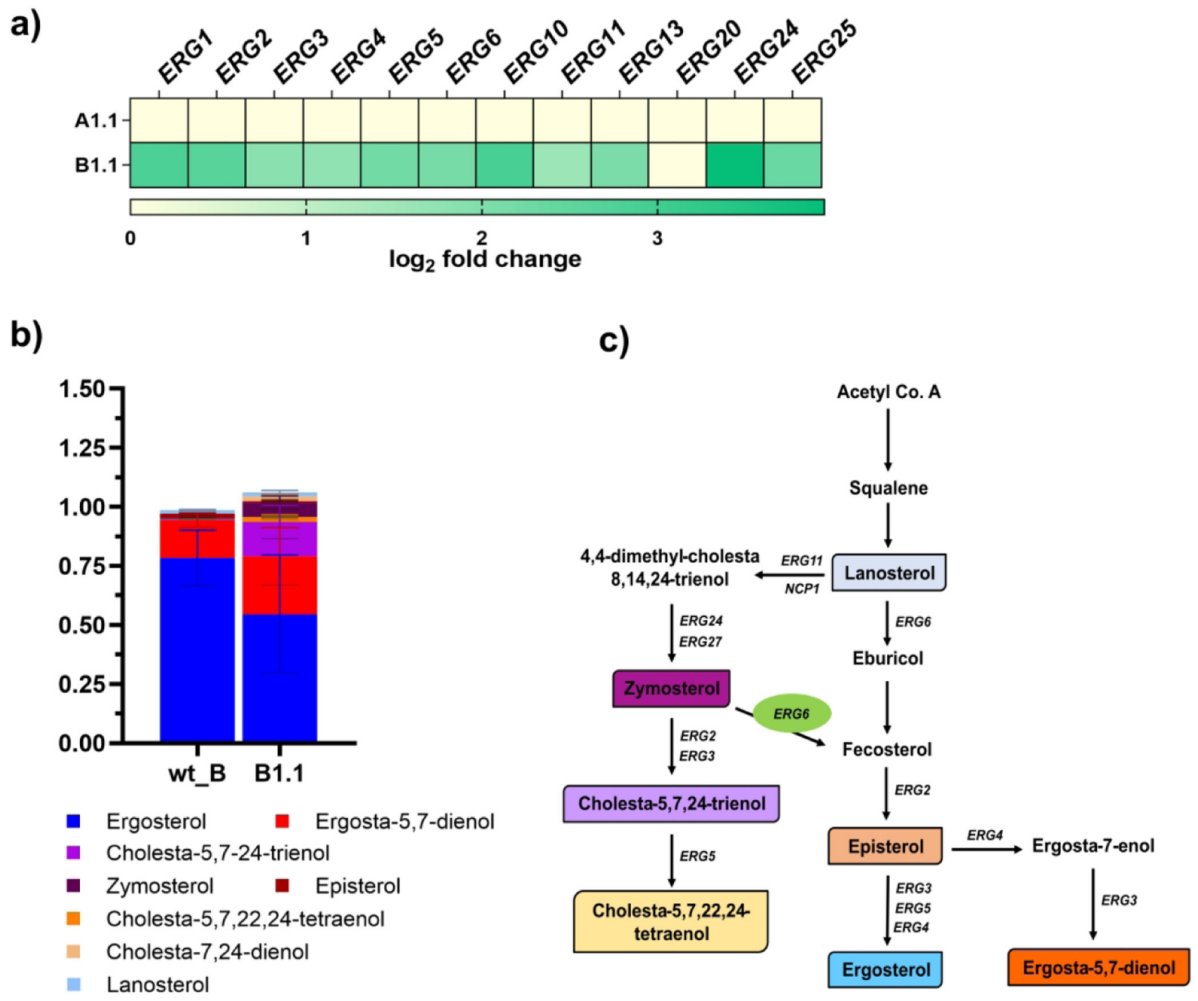
ERG genes expression and the ergosterol content in adapted strains. **a**) Heatmap depicting the differential expression levels of the ergosterol biosynthesis pathway genes observed in the RNA sequencing results of the A1.1 and B1.1 adapted strains. **b)** GC MS-based sterol profile of the B1.1 adapted strain. **c)** Predicted ergosterol biosynthesis pathway (Carolus *et al*., 2024). The colored intermediates align with the intermediate levels in **b** and **c**. Data are based on biological triplicates, with means and standard deviations (n=3) shown.

Unlike A1.1, the B1.1-adapted strain exhibited upregulation of several ERG genes, including *ERG1, ERG2, ERG3, ERG4, ERG5, ERG6, ERG10, ERG11, ERG13, ERG24*, and *ERG25* (Figure 8a). The presence of the *ERG6*^*K371N*^ SNP in the B1.1-adapted strain, along with sterol analysis, confirmed the accumulation of cholesta-5,7,24-trienol along with significant levels of ergosterol (Figure 8b and 8c), indicating its partial influence on AmB susceptibility. Notably, the A1.1-adapted strain showed no modulation in the expression of *ERG* genes or *HSX11*, suggesting that only the B1.1-adapted strain relies on lipid modulation in the evolution of AmB resistance.

### AmB-resistant B1.1 adapted strain shows compromised cell wall integrity

**Figure 9:**
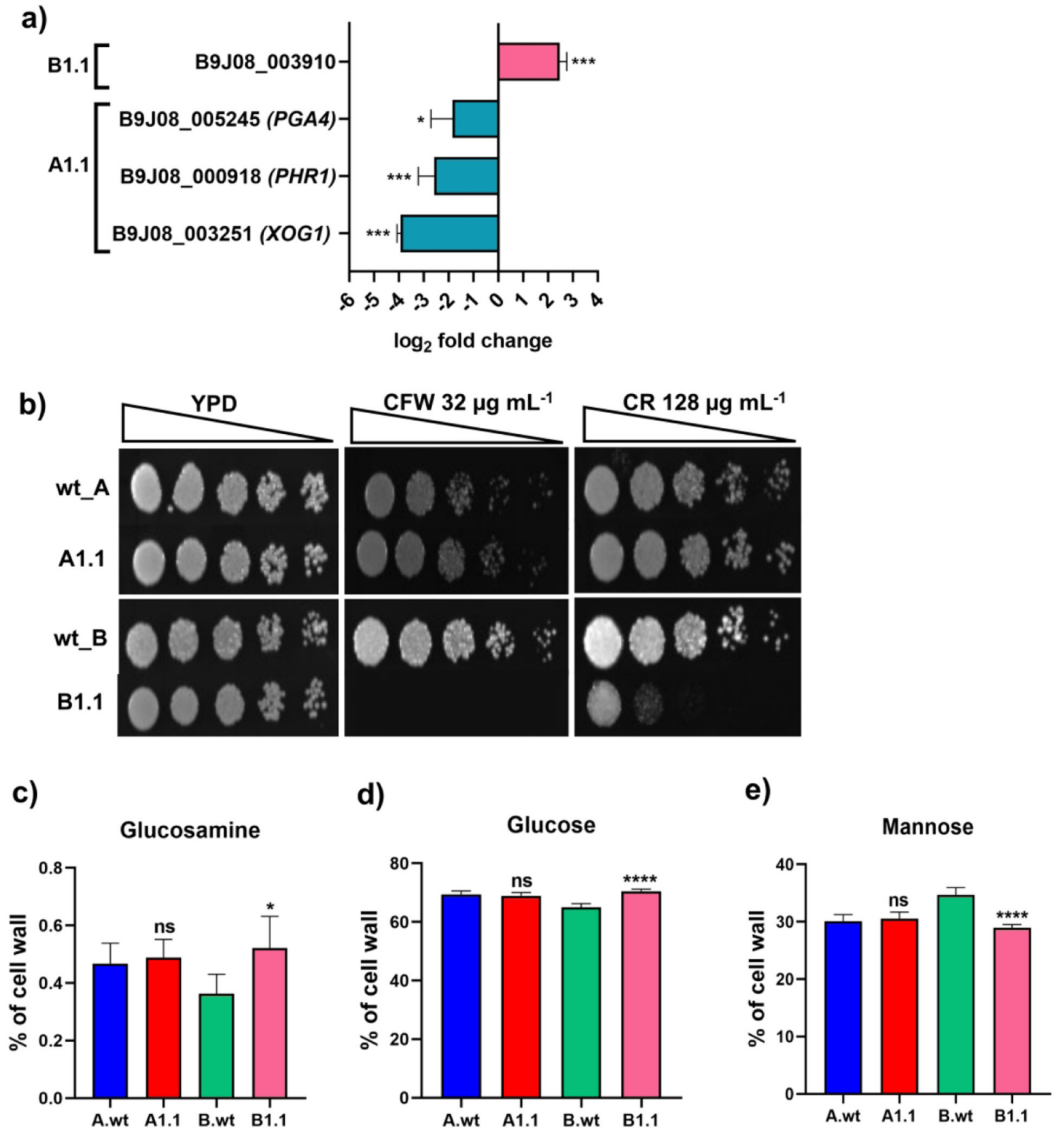
Characteristics of the CW in the A1.1 and B1.1 adapted strains. **a)** The expression of CW-related genes validated by Real-Time quantitative PCR normalized against the housekeeping gene *ACT1, LSC2*, and *UBC4*. (The list of primers used is in supplementary Table S2.) **b)** Spot assays depicting the susceptibility of A1.1 and B1.1 adapted strains towards CFW and CR. The OD_600_ of overnight grown cells was set to 0.1 and serially diluted up to five fold dilutions (represented by the gradient triangle above the spot images), which were then spotted (3 µL per spot) onto YPD plates with or without the drug. Growth differences were recorded after 48 h of incubation at 30°C by Bio-Rad XR+ Gel documentation system. **c), d), and e)** The analysis of relative polysaccharide levels in the cell wall; glucosamine, glucose, and mannose representing chitin, β-glucans, and mannans respectively in the A1.1 and B1.1 adapted strains compared to their parental strains.

The Cell Wall Integrity (CWI) is an important determinant in influencing AmB susceptibility. A study by Mesa-Arango *et al*., 2016 had reported increased levels of β-glucans in AmB resistant isolates of *C. tropicalis*. These isolates also exhibited susceptibility towards Congo Red and impacted immune response elicited by the host. Another study (Seo *et al*., 1999) reported altered CW composition in response to AmB resistance in *Aspergillus flavus*. In the present study, in RNA Seq analysis, we found several ORFs identified as orthologs of genes that are involved in cell wall (CW) biosynthesis, including *B9J08_003251 (XOG1), B9J08_005245 (PGA4)*, and *B9J08_000918 (PHR1)*, were significantly downregulated in the A1.1 adapted strain. The ORF *B9J08_003910*, which is classified as a gene with an uncharacterized function related to CW activity and localization, exhibited a >2 log_2_ fold expression in the B1.1 adapted strain. Such expression patterns suggest differences in the regulatory response of the CW biosynthesis pathway between the two adapted strains, potentially suggesting distinct adaptive mechanisms. The expression levels of the mentioned CW-related genes were validated by qRT-PCR (Figure 9a). The impact of DEGs related to CW synthesis was tested by spot assays on agar plates containing CW perturbing agents. We observed that B1.1 adapted strains displayed significantly increased susceptibility to Calcofluor White (CFW) and Congo Red (CR), while it remained unchanged in adapted strain A1.1. (Figure 9b). CFW binds to the chitin in the fungal cell wall, and CR binds to β-glucans. The chitin, β glucan, and mannan levels were determined by quantifying glucosamine, glucose, and mannose, respectively. Quantitative analysis of CW components (Figures 9c, 9d, and 9e) revealed that the level of glucosamine (a precursor of chitin biosynthesis) was significantly elevated in the B1.1 adapted strain, which also aligns well with our phenotypic analysis where cells showed increased susceptibility towards CFW, which binds to chitin. No significant changes in the CW glucans were observed for the A1.1 adapted strain (Figure 9d). The growing significance of cell wall integrity in modulating AmB susceptibility in *C. auris* warrants further investigation, which we are actively pursuing.

## Discussion

This study aimed to gain further insights into the mechanisms underlying AmB resistance in *Candida auris*. To achieve this, we selected two drug-susceptible *C. auris* strains from different geographical locations: wt_A, originally isolated from a patient's ear canal in Japan, and wt_B, recovered from an Indian diabetic patient. These strains were subjected to directed evolution by continuous exposure to sublethal concentrations of AmB to identify molecular changes that lead to resistance development.

The study identified *AOX2* as a key player in resistance to amphotericin B (AmB), as deletion of the gene resulted in an increased susceptibility in both adapted strains. *AOX2* appears to be important for developing resistance, likely due to its role in managing oxidative stress, a known factor in AmB's fungicidal action (Liu *et al*., 2005). The importance of *AOX2* became more evident when *AOX2*-deleted strains, which were initially susceptible to AmB, were subjected to experimental evolution. Unlike their progenitors, the *AOX2* knockouts could not develop resistance to AmB, underscoring the necessity of a functional *AOX2* for sustained resistance. Extensive evidence suggests that AmB's mechanism of action goes beyond just binding and depleting membrane ergosterol. AmB can undergo autoxidation, generating oxidative stress that contributes to its fungicidal effects (Vincent *et al*., 2013; Blatzer *et al*., 2015; Vriens *et al*., 2017; Posch *et al*., 2018; Guirao-Abad *et al*., 2020). Several studies have shown that hypoxia can protect the protoplasts of *C. albicans* from AmB, and enzymes like catalase and superoxide dismutase (SOD) can prevent AmB-induced cell lysis. For example, *Aspergillus terreus*, which is intrinsically resistant to AmB, has normal levels of ergosterol in its cell membrane and exhibits increased catalase levels, suggesting a catalase-based resistance mechanism that mitigates AmB-induced oxidative stress, preventing cell damage (Blatzer *et al*., 2015; Posch *et al*., 2018). Previous studies (Li *et al*., 2011; Duvenage *et al*., 2019) have also indicated that *AOX2* is involved in mitochondrial alternative respiration, mycelial development, and virulence in *Candida* species. Another study (Liu *et al*., 2005) showed that *C. albicans AOX2* and other stress response genes were upregulated in response to AmB exposure. In this study, the direct involvement of *AOX2* in AmB resistance was evident. While the deletion of *AOX2* resulted in similar phenotypes in both A1.1 and B1.1 adapted strains, transcriptional differences between the strains indicate nuanced resistance mechanisms that warrant further investigation. These findings highlight the critical role of *AOX2* in AmB resistance not only in the adapted strains but also in the clinical isolate of clade I.

The study also highlighted the impact of *UPC2* and *RTG3* in regulating *AOX2* expression and impacting AmB susceptibility. WGS of the evolved strains identified several single nucleotide polymorphisms (SNPs) associated with their adaptation and reduced sensitivity to AmB. The mid-point analysis of adaptors during the evolution experiments highlighted a strong correlation between the appearance of specific SNPs and the increased AmB MICs. The two mutations in *UPC2*^*S332R*^, *RTG3*^*S101T*^ show a direct impact towards AmB susceptibility since their restoration to WT allele also restored increased susceptibility to AmB. Interestingly, both the transcription factors, *UPC2* and *RTG3*, also regulate *AOX2* transcription. Our findings, for the first time, establish a direct link between *UPC2* and *RTG3* in regulating *AOX2* expression and AmB resistance.

The sterol analysis revealed the accumulation of cholesta-5,7,24-trienol, which can be linked to the mutation *ERG6*^*K371N*^ in the B1.1 adapted strain. Despite this mutation leading to the accumulation of sterol intermediate, significant levels of ergosterol were also observed, likely because the mutation results in a partially active protein. A partial loss-of-function point mutation in *ERG6*^E329*^ in an isolate of *C. auris* belonging to Clade IV has been previously reported (Carolus *et al*., 2024). In the same study, the evolved isolates of other clades, apart from the Clade IV, exhibited different loss-of-function mutations that were associated with fitness trade-offs. These observations suggest that a full loss of function of *ERG6* leads to fitness trade-offs that might be mitigated by a partial loss-of-function mutation. Our findings demonstrate the complex processes contributing to AmB resistance and show that the two isolates adapted to AmB through distinct pathways. While a direct role of *UPC2* in impacting AmB resistance is evident in the A1.1 adapted strain, a different mechanism involving sterol metabolism is observed in the adapted strain B1.1. Unlike the A1.1-adapted strain, the B1.1-adapted strain did not show any SNPs in *UPC2*. Instead, they exhibited perturbations in sterol and SL metabolism, characterized by an SNP in the *ERG6*^*K371N*^ gene and a significant downregulation of *HSX11*, a gene encoding glucosylceramide synthase. This resulted in a significant loss of GlcCer and an accumulation of its precursor, αOHCer. These findings suggest that the B1.1-adapted strain relies more on lipid metabolism compared to the A1.1-adapted strain. A gene related to oxidoreductase activity (B9J08_000008), an acytransferase encoding gene (B9J08_000010), and two uncharacterized genes (B9J08_000006 and B9J08_000007) were co-downregulated with the *HSX11*. These could be regulated by a common transcription factor, which may be affected during the development of resistance to AmB, which needs to be determined.

Overall, This study highlights the diverse and complex mechanisms by which *C. auris* adapts to AmB resistance. While *UPC2, RTG3*, and *AOX2* play a central role in A1.1, lipid metabolism, ergosterol pathway, and compromised CWI are more prominent in B1.1. These findings underscore the variability in resistance strategies, even within closely related strains, and open avenues for further exploration of the interplay between ergosterol, sphingolipids, oxidative stress, and AmB resistance.

## Material and Methods

### Strains and media

The Clade II reference strain, B11220 (wt_A), was obtained from the Fungal Biodiversity Centre (CBS) collection. Another Clade II susceptible clinical isolate, NCCPF 470296 (P2428) (wt_B), was sourced from the National Culture Collection of Pathogenic Fungi (NCCPF), an Indian Council of Medical Research (ICMR), New Delhi. After microevolution experiments, all these isolates and their evolved strains were kept in 30% glycerol and frozen at -80 °C. For all experiments, the strains were cultured at 30 °C in YPD medium (1% yeast extract, 2% peptone, and 2% dextrose). Table S1 contains a list of strains used in this study.

### Clade-analysis

The Clade-specific primers were used to identify the Clade status of the isolates (Narayanan *et al*., 2022b). The raw Illumina sequencing reads (deposited in NCBI database under the Bioproject PRJNA1012821) of P2428 were mapped to the GenBank assemblies GCA_002759435.1 (Clade I, strain B11205), GCA_003013715.1 (Clade II, strain B11220), GCA_016772215.1 (Clade III, strain B12037), GCA_008275145.1 (Clade IV, strain B11245), and GCA_016809505.1 (Clade V, strain IFRC 2087).

The Clade was further confirmed by phylogenetic analysis by using isolates from different Clades along with their respective reference genome assembly.

### *In vitro* evolution of *C. auris* susceptible isolates

For *in vitro* evolution, two susceptible *C. auris* strains wt_A and wt_B (MIC_50_-AmB -0.5 µg mL^-1^, and 1 µg mL^-1^) were subjected to the *in vitro* evolution procedure described in (Gerstein and Berman, 2020; Narayanan *et al*., 2022a), with some modifications. A single colony from revived cells from frozen stock was cultured in a fresh 10 mL YPD broth and was incubated for 72 h at 30°C. Samples of 10 µL of 0.1 O.D._600_ cells were transferred to three separate tubes from this stationary phase culture, with and without AmB (0.5 µg mL^-1^ and 1 µg mL^-1^ of AmB for wt_A and wt_B, respectively) to a total culture volume of 10 mL, in triplicate. Two parallel cultures without AmB were labeled as C1 and C2 as replicates of controls. A second set of replicates was continuously exposed to AmB and was labeled as A1 (adapted strain of wt_A) and B1 (adapted strain of wt_B). The culture from all the replicates and controls were diluted in a ratio of cells: media as 1:1000 dilution both with and without drug and incubated for the next 72 h at 30°C. One 1:1000 dilution of culture to media at 30°C for 72 h was calculated to give 10 generations (log_2_(1000) = 9.97) as described in (Gerstein and Berman, 2020). A total of 10 such transfers were done in the presence and absence of AmB to obtain an evolved population after approximately 100 generations. Every 10 generations, cell aliquots were harvested and stored at −80°C in 30% glycerol for further experiments.

### Growth assays

The growth assays were performed in flat-bottom 96-well plates using a Liquid Handling System by a micro-cultivation method (multimode microplate reader, Tecan, USA) in YPD media at 30°C. The cells were adjusted to an OD_600_ of 0.1 in a total volume of 200 μL in the 96-well plate. This was done for the overnight-grown *C. auris* cultures, which were divided into 2 groups, in the presence of AmB and in the absence of the drug, which served as the control group. At every 30 min intervals, the OD_600_ was measured before mixing the culture for 180 sec, and this was followed for a period of 48 h.

### Determination of Minimal inhibitory concentrations (MICs) and spot assays

MIC_50_ was determined using the broth dilution assay (BDA) following Clinical and Laboratory Standards Institute (CLSI) guidelines to assess the susceptibility to AMB (amphotericin B; Merck). A total 200µL volume of RPMI MOPS (pH 7, 2% glucose, 1% DMSO) or YPD (as indicated) medium with a final OD_600_ nm of the cells to be 0.0004 was used. A 2-fold serial dilution of the drug from 32 μg mL^-1^ to 0.125 μg mL^-1^ concentration was prepared in a round-bottom 96-well polystyrene microtiter plate (Greiner). After 48 h of incubation at 30°C, the growth of the plates was measured spectrophotometrically (OD_600_) using a BioTek SynergyTM H1 microplate reader.

For spot assays, 10^6^ cells were taken from an overnight grown culture and serially diluted to 5 -folds, and these dilutions were spotted (3 µL of spot volume) onto YPD plates with and without the drug. After an incubation of 48 h at 30°C, the growth was recorded by the Bio-Rad XR+ Gel documentation system.

### Genomic DNA isolation

Following the manufacturer's instructions, genomic DNA was extracted from the cells cultured in YPD liquid using the Qiagen Yeast DNA Kit (QIAamp DNA Mini QIAcube Kit). After that, genomic DNA was eluted with sigma water, and the Nanodrop 2000 spectrophotometer (Thermo Scientific, USA) was used to measure the concentration (absorbance at 260 nm) and purity (ratio absorbance at 260 nm/280 nm). In Bangalore, India, at Clevergene Biocorp Pvt Ltd, whole genome sequencing was carried out.

### Whole genome sequencing data analysis

Sequencing data analysis from in vitro microevolution experiments was performed using the perSVade software (v. 1.0.6)(Schikora-Tamarit and Gabaldón, 2022). Pipeline includes several modules for sequencing read trimming, quality assessment, alignment, variant calling, and the functional annotation of identified variants. Alignment of the trimmed reads was performed using corresponding *C. auris* B8441 (v. s01-m01-r27) reference genomes retrieved from Candida Genome Database (CGD). Module call_small_variants includes integrated output (SNPs and small indels) from three different variant callers: BCFtools (v.1.9), GATK Haplotype Caller (v. 4.1.2), and Freebayes (v. 1.3.1). Additional filters (read coverage below 30 and a minimum allele frequency (--min_AF) below 0.9) were applied to remove low-quality variants from the results of the variants calling. Only those variants that passed the filters of all three callers were considered as high-confidence per each sample. All the variants identified in wild-type (WT) strains were filtered out from the high-confidence sets of variants identified in evolved strains (with BCFtools v.1.15.1, function isec). The annotation of final set of variants was performed with the module annotate_small_vars (with Ensembl Variant Effect Predictor v.100.2) to determine the functional impact of each variant (missense, nonsense, frameshift, in-frame insertions/deletions, or splice region variants). Tables 12 and 3 (https://www.ncbi.nlm.nih.gov/Taxonomy/Utils/wprintgc.cgi Accessed 24/07/24) were used for Genetic and Mitochondrial Genetic code translation for *C. auris*, respectively. For this study only protein-altering variants were considered in further analysis.

Moreover, high-confidence variants detected in the studied strains were used for phylogenetic tree reconstruction and clade identification, incorporating supplementary data from strains across five *C. auris* clades (Carolus *et al*., 2023). For tree reconstruction, the method used in (Schikora-Tamarit and Gabaldón, 2022) was applied. It includes pseudo-genome sequence generation for each sample by replacing reference bases with haploid SNPs, considering only variable positions and excluding INDELs from original variants. Aligned pseudo-genomes were used to construct unrooted trees using IQ-TREE with the parameter ‘-m TEST+ASC’ for automatic model selection and ascertainment bias correction. The final tree was obtained using midpoint rooting with support values calculated from 1000 bootstrap replicates.

### Electrophoretic karyotyping

Electrophoretic karyotyping was carried out as mentioned in (Narayanan *et al*., 2022a)single-colony overnight cultures served as the inoculum for secondary cultures, which were cultivated until OD_600_= 0.9. For the chromosomal plug preparation, about 3 OD cells were employed. The manufacturer's instructions (Bio-Rad) were followed, and Cleancut agarose (0.6%), lyticase enzyme, and Proteinase K were used. Using 0.5X TBE as the running buffer, the chromosomes embedded in the agarose plugs were separated on a 1.0% agarose gel (Bio-Rad). The run protocol is as follows: switch for 60–60 sec at 6V/cm and 120° over 8 h at 12°C. Then, switch for 90–150 seconds at 6V/cm and 120° over 18 h at 12°C. The run was conducted on a Bio-Rad CHEF-DR III machine. After the run, the gel was stained with ethidium bromide, and the bands were visible thanks to the Bio-Rad Gel Documentation System.

### Allelic variant construction

Plasmid pJMR19 was digested with *Sap*I in order to reverse the SNP in the mutant (adapted) strain, and the corresponding duplexed oligos were then ligated with the digested pJMR19 plasmid. Using the NEB T4 DNA ligase enzyme in accordance with manufacturer instructions, 40 ng of the *Sap*I digested pJMR19 plasmid was combined with 100 nM of the duplexed oligos for ligation. Donor DNA was amplified using primers flanking 500 bp upstream and downstream of the SNP position. A sample of 5 µg of the matching pJMR19 construct and 5 µg of dDNA were used in the transformation mixture. The transformation method for strain construction was followed as mentioned in (Doorley *et al*., 2025). Single colonies of the corresponding adapted strain (A1.1) in which the allelic variant has to be constructed were cultured overnight at 30°C in an incubator. From this, the precultures were cultivated until the OD_600_ ranged from 1.6 to 2.2 (which is achieved in about 3–4 h) after being diluted in 50 mL YPD in a conical flask. After 5 min at 3273 x g, the cells were harvested, resuspended in 10 mL of transformation buffer (10 mM Tris-HCl, 1 mM EDTA•Na2 (VWR), and 100 mM LiOAc (Sigma), and shaken for 1 h at 30°C and 150 rpm. After adding 1M DTT (VWR) (250 µL), the cells were further incubated for 30 min. After pelleting for 5 min at 5000 x g at 4°C, the cells were rinsed twice: first with 25 mL of ice-cold dH_2_O and then with 5 mL of 1 M ice-cold sorbitol (Sigma). After carefully removing the supernatant, the pellet was again suspended in 200 µL of ice-cold 1 M sorbitol. The transformation mixture was combined with 40 µL of the *E. coli* TOP10F’ competent cells, which were then placed in a 2 mm electroporation cuvette (Pulsestar, Westburg). A single pulse was then provided at 1.8 kV, 200 W, and 25 µF, and 1 mL ice-cold sorbitol was added. The electroporated cells were pelleted and immediately transferred to 1 mL YPD in test tubes and incubated at 30°C for 3 hrs, followed by a 5 min 5000 x g centrifugation and a resuspension in 100 µL YPD and plating on YPD agar supplemented with 200 mg/mL nourseothricin (Jena bioscience). After 2 to 3 days of incubation at 30°C, transformants were visible. Positive transformants were selected for the analysis.

### Sterol extraction and estimation

The sterol analysis method was followed as mentioned by (Carolus *et al*., 2024), in which the cells grown in RPMI were cultured for 48 h at 30°C until stationary phase. After an equivalent OD_600_ of 20, cells were collected by centrifugation and then washed with 1X PBS. The cell pellets (~20 mg) were resuspended in 300 µL saponification solution (12.5 mg of KOH dissolved in 18 mL of MQ water and adjusted to 50 mL in 98% ethanol) by vortexing. The mixture was then heated in a capped glass vials for 1 h at 80°C. After cooling to room temperature, the samples were mixed with 100 µL MQ water and 400 µL hexane containing 1 µL of 5 mg/mL cholestane as an internal standard. After vortexing for 3 min, the samples were left for 20 min for phase separation, and 350 µL of the top layer was collected in a glass tube. The bottom layer was reextracted with 600 µL of hexane: 3 min vortexing followed by 20 min phase separation, and 550 µL of the upper phase was collected. The two hexane fractions were combined and allowed to dry at room temperature using vacuum centrifugation. Sterol extracts were re-dissolved in 60 µl hexane and derivatized by adding 10 µl of a silylating mixture (Sigma, 85432), short vortexing, and incubation at room temperature for at least 1 h. Derivatized extracts were shortly centrifuged to precipitate potential debris, and 50 µl of the extract was transferred to a smaller insert glass tube for GCMS analysis.

A Thermo Scientific gas chromatography-mass spectrometer (Trace 1300 - ISQ QD fitted with a Restek Rxi-5ms capillary GC column and a TriPlus RSH autosampler was used to analyse the samples. Helium was used as carrier gas at a flow rate of 1.4 mL/min. After 1 min, injection was performed in split mode at 250°C and a ratio of 1:10. The column was held at 50°C for 1 minand then the temperature was ramped up to 260°C at 50°C per minutefollowed by a ramp up to 325°C at a rate of 2°C per minute that was kept for 3 min. The mass detector was operated in scan mode (50 to 600 atomic mass units), using electron impact ionization (70 eV). The MS transfer line and detector had temperatures of 325°C and 250°C, respectively. Sterols were identified by their retention time relative to the internal standard (cholestane) and specific mass spectrometric patterns using Chromeleon software (version 7). The spectra were matched to GC-MS libraries described in (Müller *et al*., 2017), and NIST/EPA/NIH version 2.

The amounts of sterols were determined by integrating their base ion signals and comparing them to the internal standard. Sterol extraction and analysis of each strain were performed in biological triplicate.

### RNA Isolation

A saturated overnight culture was used to inoculate 10 mL of fresh YPD at an O.D._600_ of 0.1. The resulting log phase cells were cultured for 4-5 h at 30° C. Following centrifugation, the cells were collected, and DEPC water was used to wash them. The RNeasy Mini Kit (Qiagen, Hilden, Germany, Cat. No. 74104) was used to isolate total RNA in accordance with the manufacturer's instructions. Thermo Scientific, USA's Nanodrop 2000 spectrophotometer was used to measure the total amount of RNA in the samples.

### RNA Sequencing and Analysis

The sequence data was generated using Illumina NovaSeq 6000. The raw reads were processed using FastQC for quality assessment (https://www.bioinformatics.babraham.ac.uk/projects/fastqc/). TrimGalore was used to remove adapter sequences and low-quality bases (<q30) (https://www.bioinformatics.babraham.ac.uk/projects/trim_galore/). High-quality reads were then aligned to the *C. auris* B8441 reference genome (Candida Genome Database) using Bowtie2 (Langmead and Salzberg, 2012) with default parameters. Gene abundance estimates were obtained using HTSeq to calculate absolute read counts (Anders *et al*., 2015) followed by differential expression analysis with DESeq (Anders and Huber, 2010). Genes were identified as differentially expressed and classified as up-regulated or down-regulated with an absolute log2 fold change (|log2 FC|) ≥ 1. Genes that exhibit a log2 fold change of less than -1 were classified as down-regulated, those with values more than +1 as up-regulated, and those with values between -1 and +1 as neutrally regulated. Lists of all DEGs for each adapted strain are provided in Supplementary Files S1 and S2.

### Gene ontology (GO) and pathway analysis

By using the ClusterProfiler R Bioconductor package, over-representation analysis was carried out for the biological processes, molecular functions, and cellular components (Yu *et al*., 2012). GO data were retrieved via the biomaRt R package (Durinck1 *et al*., 2009). Gene Ontology (GO) terms that have a multiple test adjusted p-values below 0.05 were considered significant. GOplot R was used to visualize the GO enrichment results, with the z-score providing an overview of gene expression profiles within each GO term(Walter *et al*., 2015).

### Quantitative Real Time-PCR and analysis

Thermo Scientific, USA's Nanodrop 2000 spectrophotometer was used to quantify the total RNA that had been obtained according to the previous instructions. Following the manufacturer's instructions, about 1 µg of RNA was synthesised using the RevertAid H Minus First Strand cDNA Synthesis Kit (Thermo Fisher Scientific, Waltham, MA, United States, Catalogue No: K2562). Using the CFX96TM real-time PCR system (Bio-Rad, USA), the desired gene-specific oligonucleotide primers (Table S2) were combined with iTaq Universal SYBR Green Supermix (Bio-Rad, Catalogue No: 172-1544) to assess the quantitative expression profile following normalisation with the housekeeping genes *CauACT1, CauLSC2*, and *CauUBC4*. By figuring out the threshold cycle (CT) value of the target genes, housekeeping gene, and *CauACT1* gene, the level of gene expression was determined. Using the 2^-ΔΔCT^ technique, comparative gene expression profiles were analysed. Technical triplicates and biological duplicates were used for qRT-PCR. Table S2 provides a list of the primers utilised in the RT-PCR.

### Gene deletions

*AOX2* was disrupted using Homologous recombination strategy with a cassette, containing *NAT1* gene, which encodes for nourseothricin acetyltransferase to confer resistance to nourseothricin. The 5′- and 3′-UTR (~500 bp) of the gene, *AOX2*, were amplified separately from wild-type genomic DNA and fused with one-half each of the *NAT1* gene amplified from a plasmid. The two *NAT1*-amplified fragments shared a ~300–350-bp complementary region. The fused PCR products were co-transformed into parental (wt_A and wt_B) and adapted (A1.1 and B1.1) strains followed by plating the transformants on YPD agar plates supplemented with nourseothricin (200 µg mL^-1^) to select for homologous recombinants. Nourseothricin-resistant colonies were then picked and checked for gene deletion by colony PCRs.

### ROS estimation

A saturated, overnight culture was used to inoculate 10 mL of fresh YPD at an OD_600_ of 0.1, which was grown for 5-6 h at 30°C to obtain a log phase culture. The cells were then collected by centrifugation and washed with sterile PBS buffer. The cells were then incubated with 10 µM DCFDA (2'-7'-dichlorodihydrofluorescein diacetate) for 30 min in the dark. Post incubation, the cells were washed and resuspended in 1 mL PBS for fluorescence measurement. The excitation and emission wavelengths taken were 480 nm and 540 nm, respectively.

### Lipid extraction

Saturated overnight cultures grown in YPD were diluted to 0.1 OD_600_ in fresh medium and were grown to OD_600_ 0.8 - to 1 (mid-log phase). Approximately 5×10^8^ cells were harvested by centrifugation at 4000xg for 5 min with three biological replicates and three biological replicates. Cell pellets were washed in distilled water. Internal standards, C17 sphingosine and C17 ceramide (d18:1, 17:0) (Avanti Polar Lipids, USA), were added to each sample, and glass beads (50 mg, 0.4-0.6 mm) were used in Fastprep® (MP Biomedical) to lyse the cells. The procedures previously outlined by (Shahi *et al*., 2020; Kumar *et al*., 2021; Ali *et al*., 2024) were used to carry out the base hydrolysis and lipid extraction. Lipids that had been extracted were N_2_ dried and kept at -20°C until analysis.

### Liquid Chromatography Mass Spectrometry

As described in (Ali *et al*., 2024) the buffer used to suspend the extracted lipids was an organic buffer made of methanol, 1 mM ammonium formate, and 0.2% formic acid. The two buffers utilised in the mobile system were organic buffer and aqueous buffer (water with 2 mM ammonium formate + 0.2% formic acid). The HPLC equipped with the column received a 5 µL sample from the autosampler, and the mobile buffer was pumped at a flow rate of 0.3 mL per minute. To separate the SLs, the C8 column (Waters, USA) was utilised. SL species were detected using QTRAP® 4500 mass spectrometer (SCIEX, USA), via targeted multiple reaction monitoring (MRM) techniques.

### Liquid Chromatography Mass Spectrometry data analysis

MultiQuantTM software (ver 3.0.3) was utilised to resolve mass spectrometric chromatograms (SCIEX, USA). Internal standard normalisation was used for quantification. Each lipid species’quantity was determined as a percentage of the total SL content normalized to per mg of protein. An aliquot of 25 μL was taken from the cell lysate of each replicate for protein estimation before lipid separation. The lipid content(ng/ml) in each replicate was then normalized to the total protein(mg/ml) of that replicate. This was done to maintain homogeneity across replicates and samples. This is done routinely as a measure while normalizing lipid contents (Shahi et al., 2020; Kumar et al., 2021; Ali et al., 2024). For every analysis, three replicates of every sample were utilised. Student's t-test was used to assess the statistical significance between the data sets; a p-value of < 0.05 was deemed significant. Data bars were done with GraphPad Prism 8. Supplementary file S3 contains the entire sphingolipid profile of the B1.1 adapted strain.

### Cell wall quantification

Yeast cells were grown in YPD overnight at 30°C, and cell wall composition was analysed as described according to (Mora-Montes, 2004). Briefly, cells were pelleted by centrifugation and washed twice in water. Cells were broken using a FastPrep machine (MP Biomedicals). The homogenate was pelleted and washed with 1 M NaCl to remove proteins. The samples were then heated for 10 min at 100 °C in SDS extraction buffer (500 mM Tris-HCL [pH7.5], 2% [wt/vol] SDS, 0.3M β mercaptoethanol, 1mM EDTA) before freeze drying. The β glucan, mannan, and chitin levels were determined by quantification of glucose, mannose, and glucosamine, respectively, produced because of hydrolysis by 2 M trifluoroacetic acid at 100 °C for 3 h. The hydrolysates were analysed by high-pressure ion Chromatography (HPIC) as described by (Mora-Montes, 2004), with the following modifications. Samples of 0.4µl were injected into a Dionex carbohydrate analyser equipped with a CarboPac PA20 column (0.4x150 mm), guard column, and an ED50 Pulsed amperometric detector (PAD). The samples were eluted with a gradient of 5-100 mM at a flow rate of 0.008 ml/min for 25 min.

## Supplementary Material

Supplementary Materials

## Figures and Tables

**Table 1:** List of identified missense and nonsense SNPs detected in the adapted strains A1.1 and B1.1. The Gene IDs correspond to Gene IDs of *C. auris* B8441 reference genome deposited in the Candida Genome Database (https://www.candidagenome.org/). The gene names correspond to the annotated orthologs of these genes in *Saccharomyces cerevisiae* or *C. albicans*. The amino acid (a.a.) changes indicate the reference a.a., its position, and the altered a.a., along with their corresponding codons, highlighting the reference and altered nucleotides in uppercase letters.

Missense and Nonsense mutations in A1.1 adapted strain				
Gene ID	*C. albicans* orthologue	Amino acid change	Nucleotide change	Variant type
**B9J08_000250**	*RTG3*	S101T	aGc/aCc	Missense
**B9J08_000270**	*UPC2*	S332R	agT/agA	Missense
**B9J08_002443**	*GNP2*	D381Y	Gac/Tac	Missense
**B9J08_004602**	*OPT1*	K217I	aAa/aTa	Missense
**B9J08_000164**	*CDR1*	Y79*	taC/taA	Nonsense
**B9J08_001676**	*HRD3*	K321*	Aaa/Taa	Nonsense
**Missense mutations in B1.1 adapted strain**				
**B9J08_003310**	*GEA2*	E1229K	Gag/Aag	Missense
**B9J08_005340**	*ERG6*	K371N	aaG/aaC	Missense
**B9J08_004907**	*PRP8*	T1561M	aCg/aTg	Missense

## Data Availability

The raw reads of RNA sequencing and whole genome sequencing have been deposited in NCBI database under the Bioproject PRJNA1012821.
