# Supplementary material for: Multi-omics Analysis of Experimentally Evolved Candida auris Isolates Reveals Modulation of Sterols, Sphingolipids, and Oxidative Stress in Acquired Amphotericin B Resistance
Source: Mol Microbiol. Author manuscript; Available in PMC 2025 Jul 4. (PMC7617760; doi:10.1111/mmi.15379)
Supplement: Supplementary Materials [file EMS206235-supplement-Supplementary_Materials.pdf]

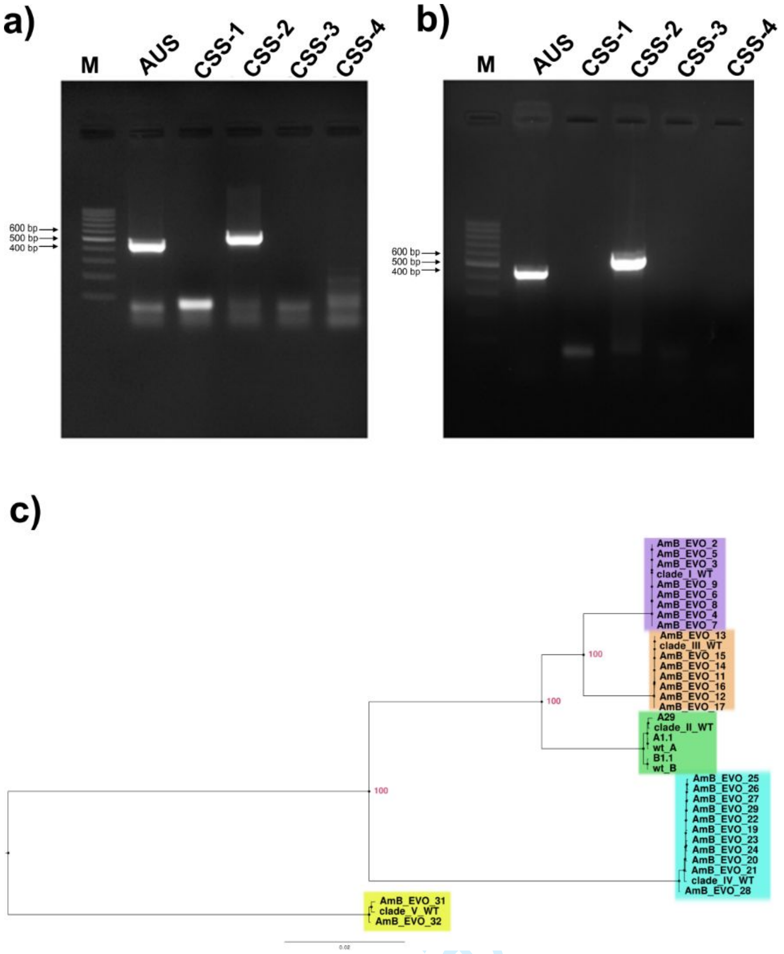

Figure S1: Clade typing for the two isolates used in this study. AUS - Auris universal sequence primers, amplify DNA sequence from four clades, CSS-I, CSS-II, CSS-III, and CSS-IV - amplicons of Clade-specific sequence for Clade I, clade II, Clade III, and Clade IV, respectively. The molecular size marker (100 bp ladder) is labelled as M. **a)** Clade typing of the strain B11220 ($CBS10913^T$), and **b)** Clade typing of the strain P2428. **c)** Phylogenetic tree depicting different isolates from five clades of *C. auris* and confirming that WT strains B11220 and P2428, along with their adapted strains, belong to Clade II. The reference strains and related isolates used in this analysis were taken from (Carolus *et al.*, 2021a; Carolus *et al.*, 2024).

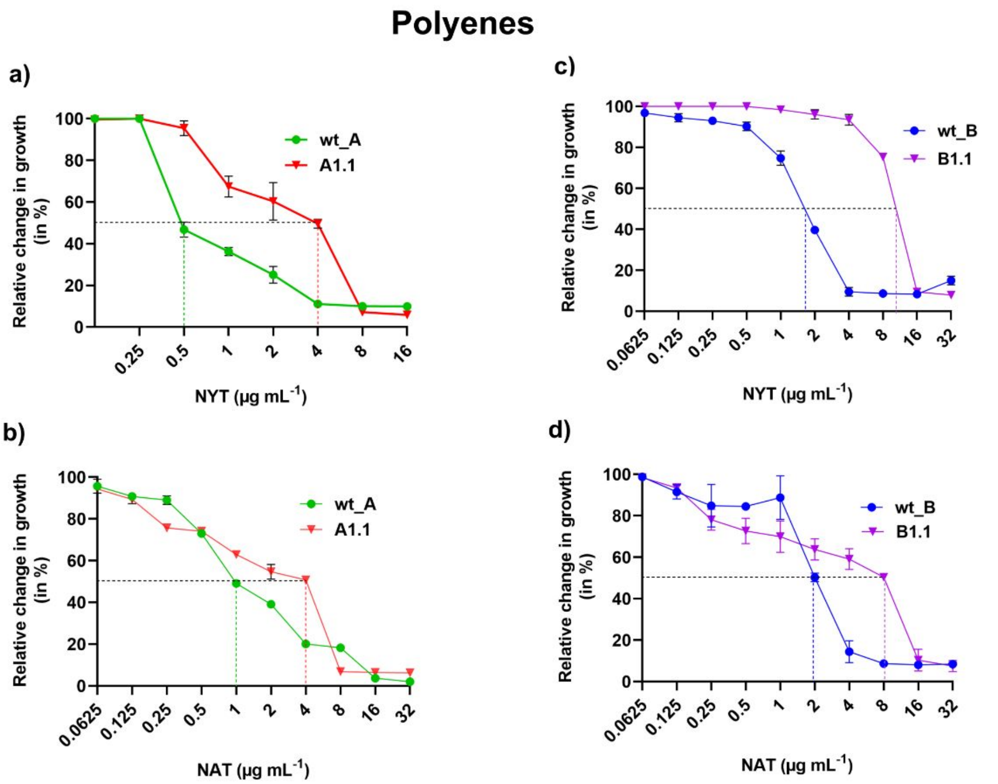

Figure S2: Cross-resistance examination of the adapted strains towards Polyenes. Susceptibility of strains A1.1 and B1.1 and their respective parental strains (wt_A and wt_B) to nystatin (NYT) and natamycin (NAT). The line plots depict drug concentrations on the x-axis and growth on the y-axis as a percentage change of growth relative to the no-drug control, after 48 h of incubation at 30°C. The growth differences were measured at OD_{600} using a Bio-Rad iMark microplate reader. The dotted lines indicate the concentration of the antifungal agent (x-axis) at which a 50% reduction of the growth (y-axis) was observed. The error bars are s.d.'s (n=3).

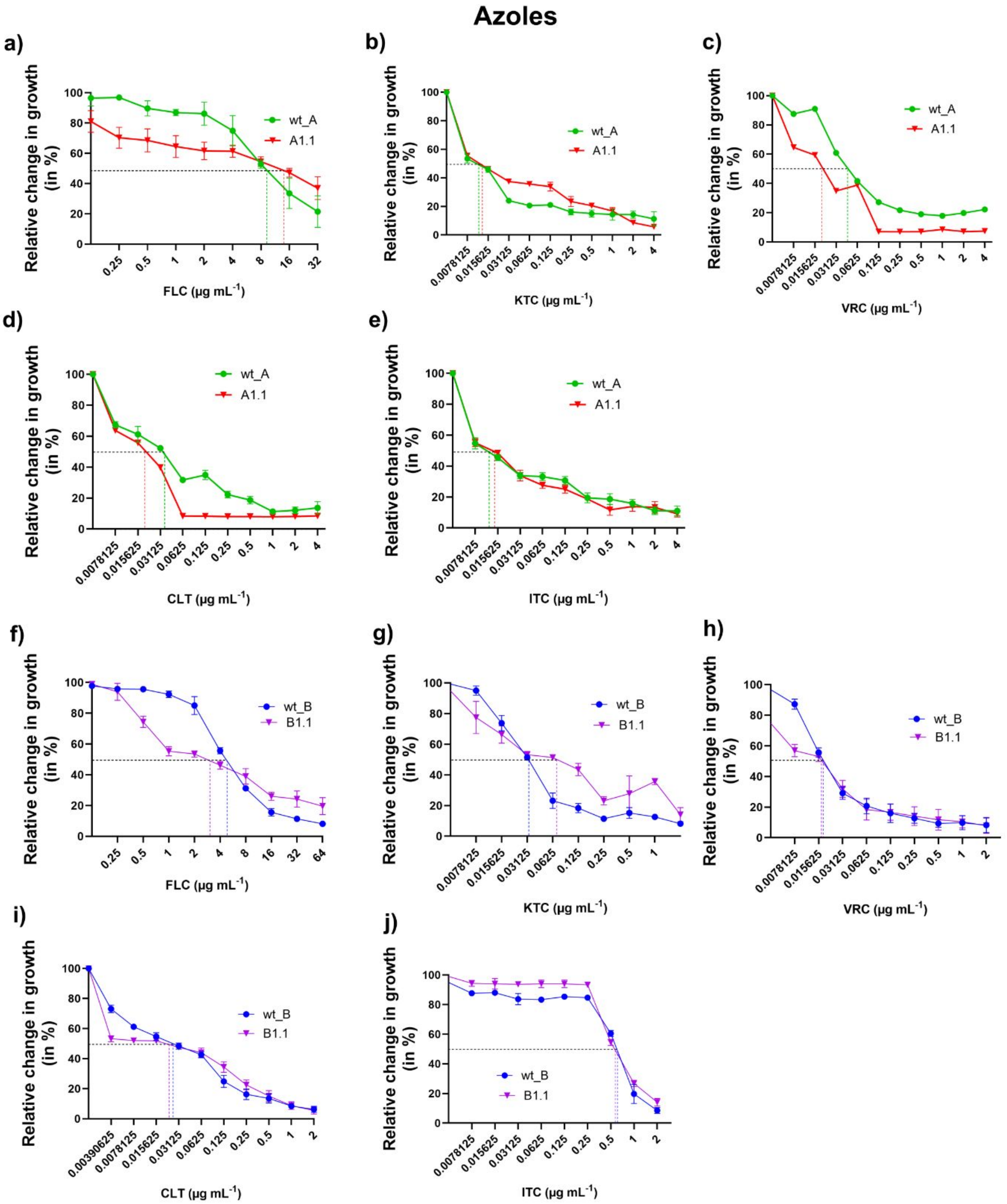

Figure S3: Cross-resistance examination of the adapted strains towards Azoles. Susceptibility of strains A1.1 and B1.1 and their respective parental strains (wt_A and wt_B) to azoles, fluconazole (FLC), ketoconazole (KTC), clotrimazole (CLT), Itraconazole (ITC), and voriconazole (VRC). The line plots depict the drug concentrations on the x-axis and growth on the

y-axis as a percentage change of growth relative to the no-drug control, after 48 hours of incubation in YPD broth at 30 °C. Growth differences were measured using a Bio-Rad iMark microplate reader. The dotted lines indicate the concentration of the antifungal agent (x-axis) at which a 50% reduction of the growth (y-axis) was observed. The error bars are s.d.'s (n=3).

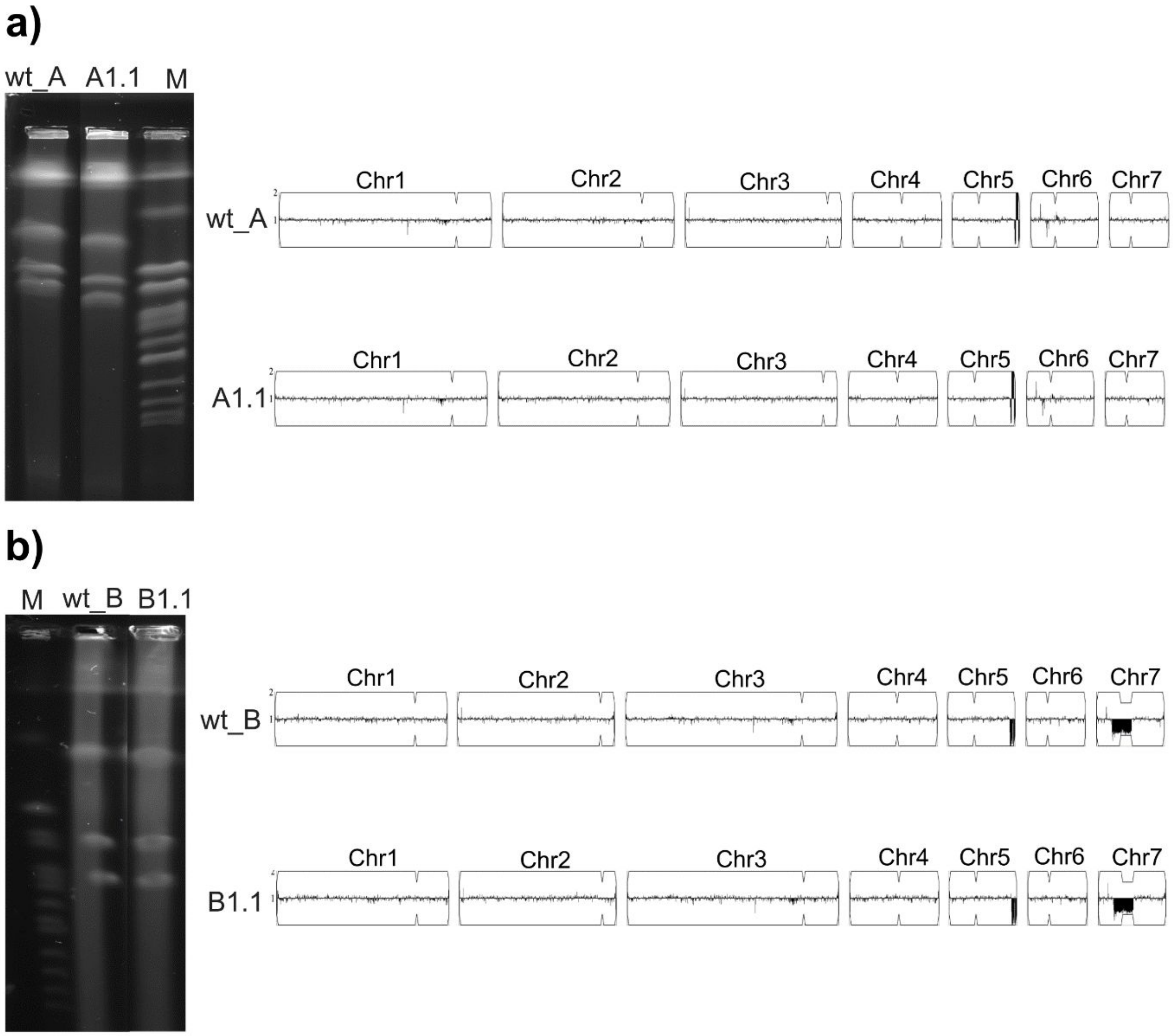

Figure S4: Analysis of ploidy in the adapted strains A1.1 and B1.1. a) Karyotype and CNV analyses of wt_A and the adapted strain A1.1 **b)** Karyotype and CNV analyses of wt_B and the adapted strain B1.1. For karyotype analysis, *S. cerevisiae* chromosomes were used as molecular size markers (M). For the CNV analysis, the scaffold numbers and the centromere locations (breaks) are shown.

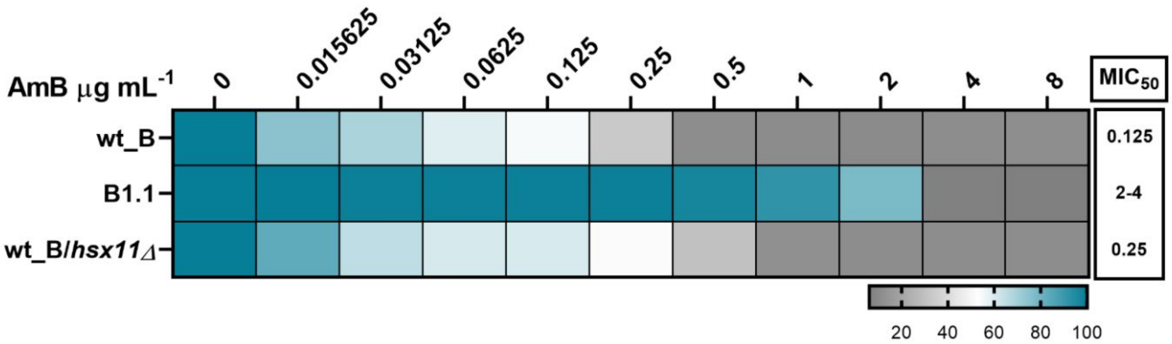

Figure S5: The antifungal susceptibility testing of *HSX11* knockout in the wt_B strain. A significant change in the susceptibility levels towards AmB were not observed in the *wt_B/hsx11Δ* strain as compared to its parental strain, wt_B.

Supplementary Tables

Table S1: Strains used in the study

Strain	Description	Source
B11220 (CBS10913^{T}) (wt_A)	Clade II type AmB susceptible strain	Central Bureau voor Schimmel Cultures (CBS), Fungal Biodiversity Centre of the Royal Netherlands Academy of Arts and Sciences (KNAW), Utrecht
A1.1	Strain obtained from the terminal line of the *in vitro* evolution experiment	This study
NCCPF 470296 (P2428) (wt_B)	Clade II type AmB susceptible strain	National Culture Collection of Pathogenic Fungi (NCCPF), Indian Council of Medical Research (ICMR), New Delhi, sponsored National facility at the Mycology Division, Department of Medical Microbiology, Postgraduate Institute of Medical Education and Research (PIGMER), Chandigarh, India
B1.1	Strain obtained from the terminal line of the *in vitro* evolution experiment	This study

For Peer Review

Table S2: List of primers used in this study.

FP (forward primer), RP (reverse primer), and RT (Real Time)

Primer name	Primer sequence (5’- - -3’)
B9J08_000486 RT FP	GAAGGAGATCACTGCTTTAGCC

B9J08_000486 RT RP	GAGCCACCAATCCACACAG
B9J08_001930 RT FP<br>B9J08_001930 RT RP	GACTCCTACTCATCGTGTTC<br>GTTCATCTCCCATCTGGTGC
B9J08_003910 RT FP<br>B9J08_003910 RT RP	CAACAGACAGTTCCAGTTTG<br>GAAGTCTCCAGAAAGACATTTG
B9J08_004469 RT FP<br>B9J08_004469 RT RP	AAGTGGTGGCTCTGTGTATT<br>GAAACCGAAGAAGGCATGTT
B9J08_000675 RT FP<br>B9J08_000675 RT RP	CAACTACACCCTAGGATCTTT<br>CCCGAAGGTATTTACGATTTC
B9J08_000438 RT FP<br>B9J08_000438 RT RP	CTGTAAAGACTTGAACGATCC<br>AGAACTTGACTCAGAAGATCC
B9J08_004476 RT FP<br>B9J08_004476 RT RP	GAATGACCCATACCACTACTTT<br>TAATAGAGGCAGATGGCTTTG
B9J08_003251 RT FP<br>B9J08_003251 RT RP	TATATAACGCCGTCTCTCTTC<br>GTCTTCAGTGTACCATGTATC
B9J08_000918 RT FP<br>B9J08_000918 RT RP	CGAATCCCAGGATAAGTATGT<br>GTACACTCTCAAGACATTGGT
B9J08_005245 RT FP<br>B9J08_005245 RT RP	GAGCTTGGAATCAACACTATC<br>CTCTAGCAATAGACGCTTTAG
B9J08_000270<br>WT_FP<br>B9J08_000270<br>SNP_RP	GTTTAGACCAATGTGTCACTCGT<br>GGGAAAGCAAAAATACGCAA
B9J08_000270<br>SNP_FP<br>B9J08_000270<br>WT_RP	CTTAGCACAACTCCCCATCG<br>CCTTAAAGCATCACCTCTACGA
B9J08_000270 MT_FP	GTTTAGACCAATGTGTCACTCGA
B9J08_000270 MT_RP	CCTTAAAGCATCACCTCTACGT
B9J08_000270 Seq._F	CCGCAGCAAGCAATCC
B9J08_001930 P1	GCCGTGACACCTCAGGCGATCC
B9J08_001930 P2	TGCGCACGTCAAGACTGTCAAGG
B9J08_001930 P3	TGTGAATGCTGGTCGCTATACTGC
B9J08_001930 P4	ATTCGTAACGCCATCACCATAGC
B9J08_001930 P5	CTTGAGACCCACCTCGCGTAGG
B9J08_001930 P6	gcgtcgacctgcagcgtacgGTTTAGATTTCTTTGTTGAAATGGG
B9J08_001930 P7	cgacggtgtcggtctcgtagCTAACGATCATAACAATGACTCAAG
B9J08_001930 P8	GACTCGGTCCCTTCTCTTATCG
B9J08_001930 P9	cgtacgctgcaggtcgacgcCTTCCGCTGCTAGGCGCGCCGTG
B9J08_001930 P10	GTCTACTACTTTGGATGATAC
B9J08_001930 P11	TCTGTTCCAACCAGAATAAG
B9J08_001930 P12	ctacgagaccgacaccgtcgGGCCGCTGACGAAGT
B9J08_001930 P13	GACTCCTACTCATCGTGTTC
B9J08_001930 P14	GTTCATCTCCCATCTGGTGC

For Peer Review

Table S3: List of gRNAs used in SNP reversal in CRISPR-Cas9 approach

Primer name	Primer sequence (5’- - -3’)
B9J08_000270 WT_TOP<br>B9J08_000270 WT_BOTTOM	CCACTCTATGACTAGTGACACAT<br>AACATGTGTCACTAGTCATAGAG
B9J08_000270 MT_TOP<br>B9J08_000270 MT_BOTTOM	CCACTCTATGTCTAGTGACACAT<br>AACATGTGTCACTAGACATAGAG
B9J08_000250 WT_TOP<br>B9J08_000250 WT_BOTTOM	CCAAATTCAAAGGTGCTGAAGTA<br>AACTACTTCAGCACCTTTGAATT
B9J08_000250 MT_TOP<br>B9J08_000250 MT_BOTTOM	CCAAATTCAAAGGTGGTGAAGTA<br>AACTACTTCACCACCTTTGAATT
B9J08_002443 WT_TOP<br>B9J08_002443 WT_BOTTOM	CCAGCTCTGTCTGTGTAGTCAA<br>AACTTGACTACACAGACAGAGC
B9J08_002443 MT_TOP<br>B9J08_002443 MT_BOTTOM	CCAGCTCTGTATGTGTAGTCAA<br>AACTTGACTACACATACAGAGC

For Peer Review
